# Modeling the effectiveness of targeting Rift Valley fever virus vaccination using imperfect network information

**DOI:** 10.3389/fvets.2023.1049633

**Published:** 2023-06-29

**Authors:** Tijani A. Sulaimon, Gemma L. Chaters, Obed M. Nyasebwa, Emanuel S. Swai, Sarah Cleaveland, Jessica Enright, Rowland R. Kao, Paul C. D. Johnson

**Affiliations:** ^1^The Roslin Institute, University of Edinburgh, Easter Bush Campus, Midlothian, United Kingdom; ^2^Royal (Dick) School of Veterinary Studies, University of Edinburgh, Easter Bush Campus, Midlothian, United Kingdom; ^3^School of Biodiversity, One Health and Veterinary Medicine, University of Glasgow, Glasgow, United Kingdom; ^4^Institute of Infection and Global Health, University of Liverpool, Liverpool, United Kingdom; ^5^Global Burden of Animal Diseases (GBADs) Programme, University of Liverpool, Liverpool, United Kingdom; ^6^Veterinary Council of Tanzania, Ministry of Livestock and Fisheries, Dodoma, Tanzania; ^7^Department of Veterinary Services, Ministry of Livestock and Fisheries, Dodoma, Tanzania; ^8^School of Computing Science, University of Glasgow, Glasgow, United Kingdom; ^9^School of Physics and Astronomy, University of Edinburgh, Edinburgh, United Kingdom

**Keywords:** Tanzania, livestock networks, network measures, meta-population model, Rift Valley fever, targeted vaccination, imperfect information, robustness

## Abstract

Livestock movements contribute to the spread of several infectious diseases. Data on livestock movements can therefore be harnessed to guide policy on targeted interventions for controlling infectious livestock diseases, including Rift Valley fever (RVF)—a vaccine-preventable arboviral fever. Detailed livestock movement data are known to be useful for targeting control efforts including vaccination. These data are available in many countries, however, such data are generally lacking in others, including many in East Africa, where multiple RVF outbreaks have been reported in recent years. Available movement data are imperfect, and the impact of this uncertainty in the utility of movement data on informing targeting of vaccination is not fully understood. Here, we used a network simulation model to describe the spread of RVF within and between 398 wards in northern Tanzania connected by cattle movements, on which we evaluated the impact of targeting vaccination using imperfect movement data. We show that pre-emptive vaccination guided by only market movement permit data could prevent large outbreaks. Targeted control (either by the risk of RVF introduction or onward transmission) at any level of imperfect movement information is preferred over random vaccination, and any improvement in information reliability is advantageous to their effectiveness. Our modeling approach demonstrates how targeted interventions can be effectively used to inform animal and public health policies for disease control planning. This is particularly valuable in settings where detailed data on livestock movements are either unavailable or imperfect due to resource limitations in data collection, as well as challenges associated with poor compliance.

## 1. Introduction

Infectious diseases are an important challenge facing livestock production systems (1) particularly in developing countries, due to their substantial impact on livestock health and welfare, and in terms of economic losses (2, 3). Worldwide, infectious diseases of animals that affect humans (zoonoses) are responsible for over 2.5 billion cases of illnesses in humans, with an estimated 2.7 million deaths every year (4, 5). Due to the impacts of many factors—including climate change, urbanization, globalization, changing eating habits, deforestation, and human-wildlife interaction—human and animal populations are at high and increasing risk of zoonotic disease transmission, emergence, and re-emergence (6–8).

Infectious diseases, including zoonoses, can spread within and between livestock populations by various routes, including direct contact, via vectors such as mosquitoes, consumption of contaminated animal products, and livestock movements (9). Livestock movements, for trading and grazing, play a significant role in disease spread between populations (10). For instance, the 2001 foot-and-mouth disease (FMD) epidemic in the UK was primarily driven by long-distance movements of livestock between holdings and local transmission within holdings at the early stage of the epidemic before a movement ban was implemented (11, 12).

Vaccination is an effective measure for controlling infectious disease outbreaks (4). However, to design an efficient vaccination strategy for infectious disease control, it is crucial to understand the behavior of disease transmission within and between populations. Decisions about where to impose a disease control strategy rely on a range of factors, including the specific pathogen, the outbreak type (whether it is a common source or propagated outbreak), the size of the target population, the contact network connecting the population, the resources available, and the effectiveness of the control strategy. While many researchers have used compartmental models that assume a well-mixed homogeneous population to explore the impact of interventions (13, 14), these models overlook contact pattern heterogeneity, which is known to significantly impact disease dynamics (13, 15–17). Network-based approach represents an intuitive way of explicitly capturing contact pattern heterogeneity (18). It is a way of describing the interplay between infectious disease transmission and the contact network pattern, as well as providing a means of testing the impact of interventions *in silico* (14, 19).

Tanzania is one of the countries in East Africa where infectious diseases, including zoonoses, pose a challenge to livestock and human health and welfare. Several efforts have been made to reduce the burden of dangerous zoonotic diseases, including rabies, Rift Valley fever (RVF), and brucellosis in Tanzania (20). For example, in 2010, the Tanzanian government launched a nationwide vaccination campaign against rabies, following many other countries aiming to meet the global target for eliminating human deaths caused by rabies by 2030 (21). As resources are often limited in low-income countries such as Tanzania, insights from modeling analyses can help guide such vaccination campaigns to yield the greatest impact on disease outbreaks. During the last officially reported RVF outbreak in Tanzania (2007–2008), extensive control measures were implemented by the government, including RVF surveillance, animal movement restrictions, ban on cattle slaughter, and vaccination of livestock (22). These measures incurred a cost of approximately US*$*3.84 million, with a significant portion allocated to livestock vaccination. Over 4 million livestock were vaccinated, primarily in areas without the disease, to prevent its further spread (23). The commercial vaccine used against RVF in domestic ruminants in Tanzania and other African countries is the live Smithburn vaccine (24), developed by Smithburn (25). While it provides long-lasting immunity with a single dose Sindato et al. (24), it can cause problems like abortions in pregnant animals and fetal malformation (26). To overcome these challenges, scientists have been developing new RVF virus vaccines (27, 28). A promising candidate is the RVF virus Clone 13 vaccine, which has undergone trials and is now approved for use in cattle and small ruminants in South Africa, Botswana, and Namibia (29).

Livestock are typically managed in distinct units, which in Tanzania comprise herds and flocks that are kept in households or multi-family compounds within villages. These livestock units are linked by movements, many of which go through markets (30, 31), forming a complex network of nodes (representing populations of livestock) and links (representing livestock movements between populations). Such networks can be modeled using a meta-population framework (32, 33), which allows us to test the impact of targeted vaccination strategies (14).

Targeted vaccination strategies that exploit the hierarchy of nodes' connectivity in a network are highly effective when the complete structure of the network is known (19, 34–36). Previous research has extensively investigated the impact of network-based vaccination strategies on an epidemic spread, primarily focusing on either theoretical (model) networks (34–40) or real-world networks from developed countries with rich livestock movement data (10, 41–44). However, the application of network structures to study vaccination strategies in African livestock settings has been limited (31, 45–48). For example, Hébert-Dufresne et al. (40) compared the effectiveness of targeted immunization strategies on various networks, including the World Wide Web, US power Grids and Co-authorship networks using SIR and SIS models. Similarly, Eames et al. (49) studied preventive vaccination on a weighted contact network using a SIR model, while Keeling et al. (42) compared pre-emptive vaccination with a combination of reactive vaccination and culling strategies for foot-and-mouth disease in the UK using a farm-based simulation model. Chaters et al. (31) analyzed livestock trade movement networks in northern Tanzania and evaluated the effectiveness of network-derived interventions, including vaccination and movement bans.

In most real-world scenarios, it is often not possible to obtain complete network data. Our knowledge of the network structure of populations is usually imperfect due to incomplete or unreliable data (50, 51). Network studies rely on data that differ from the true network connecting the population under investigation (51). Such error can substantially impact network measures, which might considerably affect their performance in epidemic control on the true network (51, 52), therefore motivating analyses to understand how different network measures are impacted by imperfect information. While studies have explored the robustness of centrality measures to missing or sampled data, and measurement errors (51–55), only a few have examined the impact of incomplete network information on the effectiveness of intervention strategies (56, 57), with limited or no research conducted using data from Africa where resource limitations and poor compliance often result in imperfect data.

In this study, we investigate the effectiveness and robustness of various vaccination strategies under imperfect knowledge of network structure. To do this, we simulated the spread of an infectious disease with properties informed by the characteristics of RVF virus spread. We use a stochastic Susceptible-Exposed-Infected-Recovered (SEIR) model on both theoretical networks (generated by simple network models, including random, scale-free and small-world) as well as a network of livestock movements across three regions in northern Tanzania (31), upon which we tested the impact of simulated targeted vaccination. Theoretical networks were included to test the robustness of our conclusions to network structure. We focused on RVF as an exemplar because it is an important vaccine-preventable livestock infection where we can build on previous studies of the role of livestock movements in the spread of infection (31). The primary goals of this study are twofold: first, to model the effectiveness of epidemic control using network-informed vaccination strategies, and second, to investigate how the performance of these strategies varies under imperfect knowledge of the network.

## 2. Methods and materials

### 2.1. Overview

This *in silico* study followed five basic steps: (1) generate a cattle movement network; (2) simulate the condition of imperfect information about the network; (3) use different network-targeted strategies to select nodes for vaccination under imperfect network information; (4) simulate multiple scenarios of Rift Valley fever (RVF) virus transmission over the network with selected vaccinated nodes; and (5) compare mean outbreak size after one year under various vaccination strategies and levels of imperfect network information. We begin this section by describing the study area and how cattle movement networks were generated, followed by a description of the network-based vaccination targeting strategies (using degree, betweenness, and PageRank centrality measures) and risk-based strategies (the risk of RVF introduction into the cattle population, derived from an index for indicating rainfall) that were considered in this study. The last three subsections focus on the RVF virus transmission model, how vaccination was implemented, and how scenarios of imperfect network information were modeled.

### 2.2. Study area

**Figure 1:**
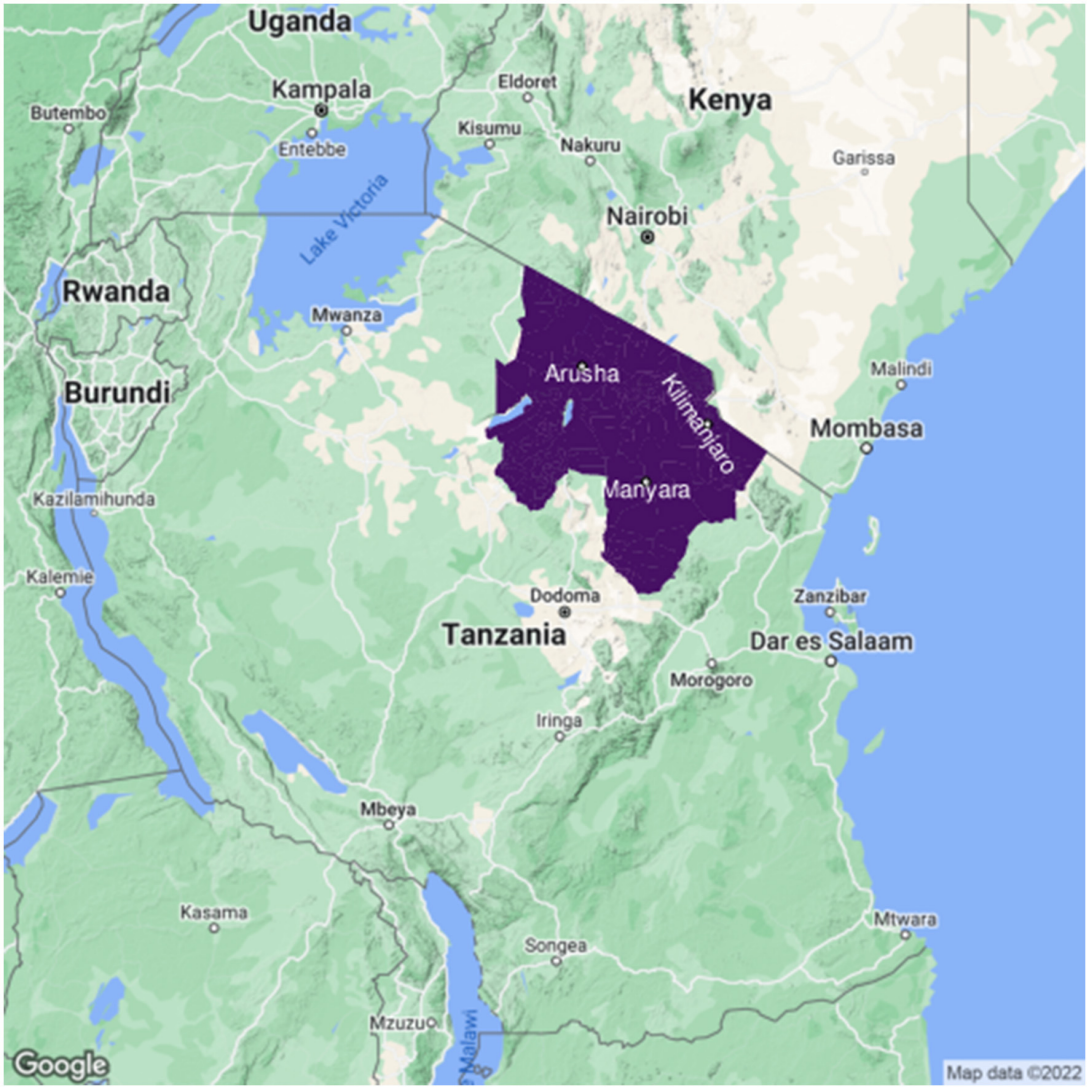
Map of the study area in northern Tanzania. Arusha, Manyara, and Kilimanjaro regions are highlighted in purple. Map created using Google map data available through ggmap package (62) in R. Shape files obtained from NBS, United Republic of Tanzania https://www.nbs.go.tz/index.php/en/census-surveys/gis.

The study area comprises three regions—Arusha, Manyara, and Kilimanjaro—located in the northern part of Tanzania (Figure 1), containing ~4.8 million inhabitants in a total area of 95,348 km^2^ (58). Livestock production in these regions is predominantly carried out for food, income, and social status (30, 31). A detailed description of the study area and its livestock practices is given in de Glanville et al. (30). There are various complex socio-economic reasons for livestock movement in northern Tanzania, including access to natural resources such as grazing, water, and salt (59), exchange of livestock as gifts and payments (60), and trade movements through the market system (31, 61).

### 2.3. Network generation

**Figure 2:**
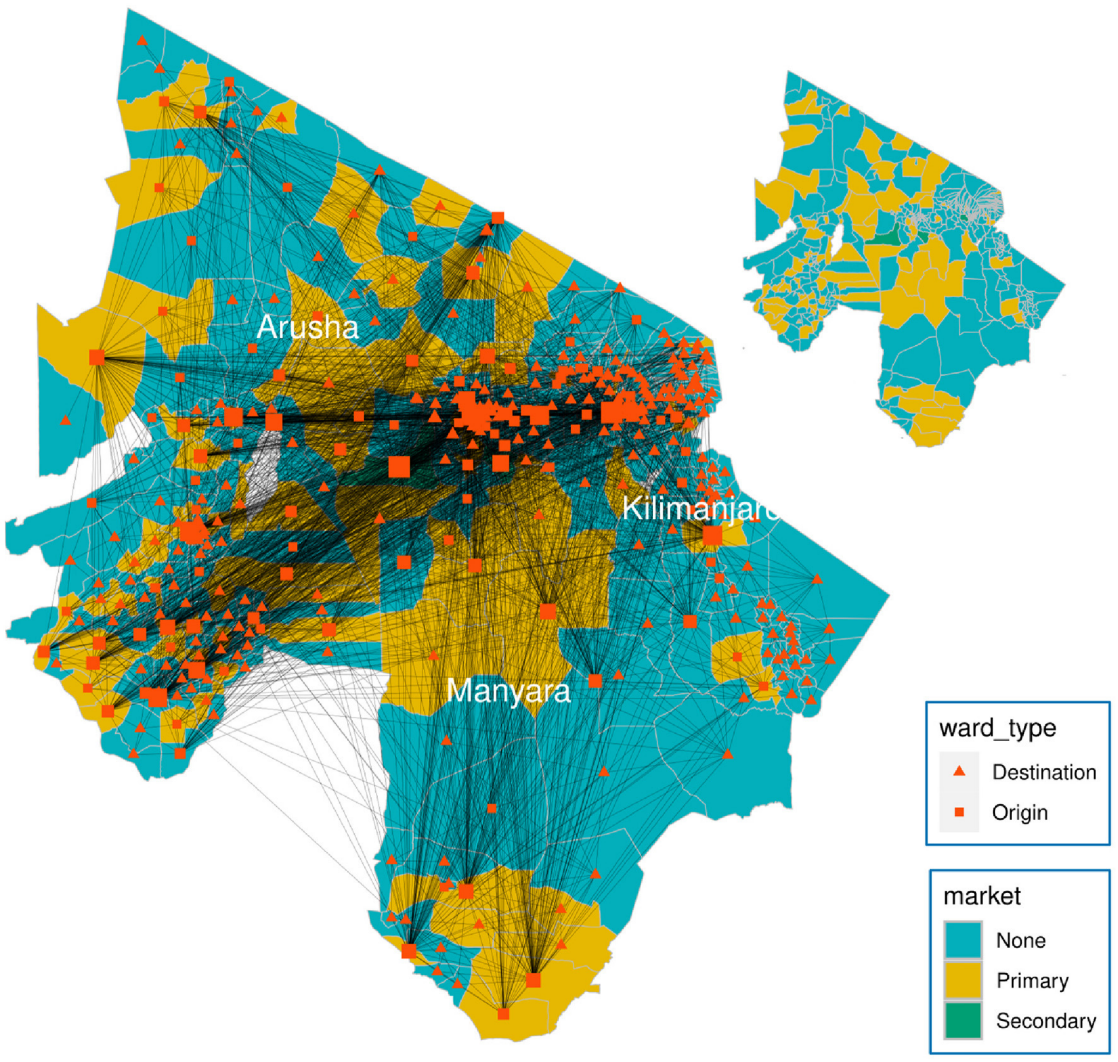
Weighted multiplex network of livestock movements between 398 wards (nodes) in northern Tanzania. Node area is proportional to node degree. Generally, cattle purchased in primary markets are batched into larger groups by traders and transported to secondary markets, where prices are higher. A node is either an origin, a destination, or both. An origin is a node with at least one outward movement of cattle via market. Movements of cattle are predominantly through primary or secondary markets, with secondary markets having the largest volume of trade (weighted degree of nodes). In addition, each ward is connected to adjacent wards through local movements of cattle, representing non-market movements such as private sales and gifting, which were not captured by the movement permit data.

This study exploits the multiplex network of cattle movements in northern Tanzania generated by Chaters et al. (31). The multiplex (which we shall refer to here as the “data-driven" network, see Figure 2) contains two layers: a movement network of cattle through markets and a network connecting adjacent wards. In the first layer, Chaters et al. (31) market movement permit data were used to generate a static network of cattle movements between 398 wards within three regions (Arusha, Kilimanjaro, and Manyara) in northern Tanzania, where the number of cattle moved is based on the estimates of the number moved in a month. A *ward* is an administrative unit of a mean area 243 km^2^ containing a mean human population of 12,000 and a mean cattle population of 9,000 across all 398 wards (31). The market permit data were collected as part of the SEEDZ (Social, Economic and Environmental Drivers of Zoonoses in Tanzania) project, the protocols and procedures of which were approved by the ethics review committees of the Kilimanjaro Christian Medical Center (KCMC/832) and National Institute of Medical Research (NIMR/2028) in Tanzania, and in the UK by the ethics review committee of the College of Medical, Veterinary and Life Sciences at the University of Glasgow (39a/15). Approval for study activities was also provided by the Tanzanian Commission for Science and Technology (COSTECH) and by the Tanzanian Ministry of Livestock and Fisheries, as well as by regional, district, ward and village-level authorities in the study area.

The cattle movement network data were aggregated spatially at the ward level and temporally by month because the destinations recorded on the movement permits could not typically be located at a finer scale (31). A spatial network, created by connecting each ward to all its spatially adjacent wards, was added to the market movement network, as a means of accounting for contacts that occur between wards through sharing of grazing and water sources, and movements of animals through gifting and private sales (31). We used the combined monthly market movement networks to generate a static directed weighted network over a year, which was then used to calculate network measures. The spatial network was excluded from the calculation of network measures because we expect targeting strategies would be guided by market network data in real policy situations.

We generated unweighted theoretical networks with an equal number of nodes (398) and mean degree (4) as the monthly “unweighted mean market movement" network to explore the effect of varying network structures on results. These included Erdős-Rényi (ER) random networks, scale-free networks, and small-world networks. ER random networks are characterized by randomly connecting nodes with a given probability, representing a baseline structure where connections are distributed with equal probability across the network (63). Scale-free networks, on the other hand, follow a power-law distribution of node degrees, meaning that a few nodes (called hubs) have many connections, while most nodes have relatively fewer connections (38). Small-world networks exhibit both local clustering and short average path lengths (64). This means that while nodes tend to be connected to their immediate neighbors due to local clustering, there are also shortcuts that connect distant nodes, allowing for a relatively rapid spread of diseases across the network (65).

Only the information on whether there was a market movement of cattle between wards in the data-driven network was used to generate the unweighted mean market network. For random networks, we use the 66. Erdős and Rényi (66) *G*(*N, M*) model with *N* = 398 nodes and *M* = 2 × 398 links, resulting in random networks with mean degree 4.

To generate small-world networks, we followed the model proposed by Watts and Strogatz (64). First, arrange nodes (wards) in a 1-dimensional circular lattice (a ring) such that each node is connected to two nearest neighbors on either side. With probability *p* = 0.1 (which is above the percolation threshold), each link is rewired, resulting in long-distance links (shortcuts) (64).

For scale-free networks, we adapted the algorithm described by Albert and Barabási (38). We begin with a fully-connected network generated using the preferential attachment model, with *N* = 398 nodes, *m* = 2 (the number of links added at each time step), and power = 1. The giant strongly connected component (GSCC) size of networks created by this algorithm is generally 1. To obtain networks with a larger GSCC size, we reshuffled the network's links while preserving the degree distribution. The summary statistics of networks generated are presented in Table 1. All networks are directed and were generated using the “igraph” package (67), available within R version 4.1.1 (68).

**Table 1:** Summary statistics of the networks generated.

**Network**	***N***	**〈*k*〉**	**Std (deg)**	**CC**	**GWCC**	**GSCC**
Erdős-Rényi	398	4	1.99	0.01	390	253
Scale-free	398	4	7.22	0.02	398	295
Small-world	398	4	0.87	0.25	398	389
Data-driven^†^	398	14.5	11	0.30	398	398

N, number of nodes; 〈*k*〉, mean degree; Std (deg), the standard deviation of network degree; CC, mean clustering coefficient; GWCC and GSCC, giant weakly and strongly connected components.

^†^The data-driven network is weighted. Its unweighted version has a mean degree of 4, like the theoretical networks, which are unweighted.

### 2.4. Network measures

In a spreading process, the importance of a node in a network is characterized by its structural position, and its contribution to epidemic spread over the network (69). Highly central or influential nodes are likely to infect or expose many other nodes disproportionately, and potentially drive the speed and severity of epidemic outbreaks (70, 71). Identifying the most central nodes is essential for breaking the transmission chain and slowing down the speed at which an epidemic is spreading (34–36). Here, we study three standard centrality measures (degree, betweenness, and PageRank) widely considered to be relevant to disease spread (34, 35, 40, 72–74).

The degree centrality of a node in an undirected network is the number of links connected to the node; for directed networks, this may be out-degree or in-degree (75). The degree centrality is vital in studying infectious disease transmission because it measures the number of potentially infectious contacts (14, 36, 75). Betweenness centrality measures the extent to which a node lies on the shortest path connecting other nodes of the network (76). In the context of disease spread, it describes the importance of a node to disease propagation across the communities of the network (14). PageRank is a metric used to rank web pages in the Google search engine (77). It ranks web pages based on the number of backlinks (number of links pointing to the page or in-degrees) or highly important backlinks (77). It indicates the probability of visiting a node by a random walker in a network (77). Each of these measures assigns “importance" differently and has their strengths and shortcomings.

### 2.5. Risk of RVF introduction into cattle population

Mosquitoes are both reservoirs and vectors for the RVF virus and, therefore, capable of keeping the virus in the enzootic cycle for a very long time—even in the absence of livestock—through vertical transmission from infected adult female mosquitoes to their offspring (78). Mosquito-borne diseases such as RVF are susceptible to climate-mediated changes (22, 79). Abnormally high rainfall can trigger the hatching and amplification of mosquito vectors, hence provoking outbreaks (80). Several studies have demonstrated that the remotely sensed Normalized Difference Vegetation Index (NDVI), one of the most used indices for green vegetation, is a good indicator of rainfall and conditions suitable for the emergence of RVF (22, 81, 82). According to Linthicum et al. (83), it is possible to anticipate RVF outbreaks in East Africa up to 5 months before they occur using a range of climate indices, including NDVI.

To calculate RVF introduction risk, the average monthly NDVI raster for the study area between January 2000 and December 2017 was divided by wards into grid cells. The risk score of a ward in the data-driven network is the proportion of cells of the ward that has an NDVI value between 0.15 and 0.4 (an indicator of regions with high rainfall, roughly equivalent to mean annual rainfall between 100 and 800 mm) (82). In theoretical networks, risk scores were randomly distributed.

### 2.6. Disease simulation

An SEIR model was used to describe the transmission dynamics of RVF virus within wards, based on Métras et al. (84). At any time, each bovid belongs to one of the four states: susceptible (S), exposed (E), infectious (I), or recovered (R). Susceptible individuals get infected and moved to the exposed class at rate β. Exposed individuals become infectious at a rate α and recover at a rate γ. We modeled transmission via vectors as delay in infectiousness onset through the state E as a large-spatial-scale approximation for the vector incubation stage and the latent period in cattle (84). To account for heterogeneity in transmission rates between wards, the average observed NDVI was incorporated into the transmission parameter, represented by the equation:


$$\begin{array}{l} R_{0}^{\left(i\right)}=\frac{\beta }{\gamma }\times \frac{\left(1-{\text{NDVI}}_{i}\right)}{\text{mean}\left(1-{\text{NDVI}}_{i}\right)}, \end{array}$$

where $R_{0}^{\left(i\right)}$ is the ward-specific basic reproduction number defined as the expected number of secondary infections produced by an infected individual in a wholly susceptible population (13). At the mean value of NDVI, the average *R*_0_ value corresponds to $\frac{\beta }{\gamma }=4$. The distribution of *R*_0_ values across all 398 wards can be found in the Supplementary Figure 6.

Because it would be unrealistic to assume homogeneous mixing of cattle within a ward, each ward was divided into 64 grid cells in an 8 × 8 matrix of sub-nodes, with each cell representing a sub-node, i.e. a sub-village within each ward (see Supplementary Figure 1 for a pictorial description of the model). Within each cell, transmission is driven by a homogeneous mixing SEIR model. Transmission between proximal sub-nodes within each ward was allowed through a spatial coupling process to account for the risk of infection transmission between sub-nodes through interactions such as the use of shared resources. The proportion of infections produced in a sub-node *i* of a ward *k* through the coupling process at any time *t* is defined by


$$\begin{array}{l} \underset{j\in N\left(i\right)}{\sum }\frac{\beta_{k}S_{i}I_{j}}{N_{j}}C, \end{array}$$

where N(i) is the set of the first neighbors of the sub-node *i*, *N*_*j*_ is the total number of cattle in a neighboring sub-node *j* of sub-node *i*, and *C* is the coupling strength. Parameter values are presented in Table 2.

**Table 2:** Model parameters used in the SEIR model.

**Parameter**	**Description**	**Unit**	**Value**	**Range**	**References**
α	Incubation period	Day	7	(5, 14)^†^	(84, 85)
γ	Infectious period	Day	7	(1, 5)	(78, 85)
*R*_0_	Mean basic reproduction number	—	4	(1, 6.8)	(84, 86, 87)
*C*	Coupling strength	—	0.02	—	User defined
*e*	Vaccine efficacy	—	1	(0.7, 1)	(29, 88, 89)

^†^The range of values for the incubation period accounts for (4, 8) days extrinsic incubation period in vectors and (1, 6) days latent stage in livestock (84).

**Figure 3:**
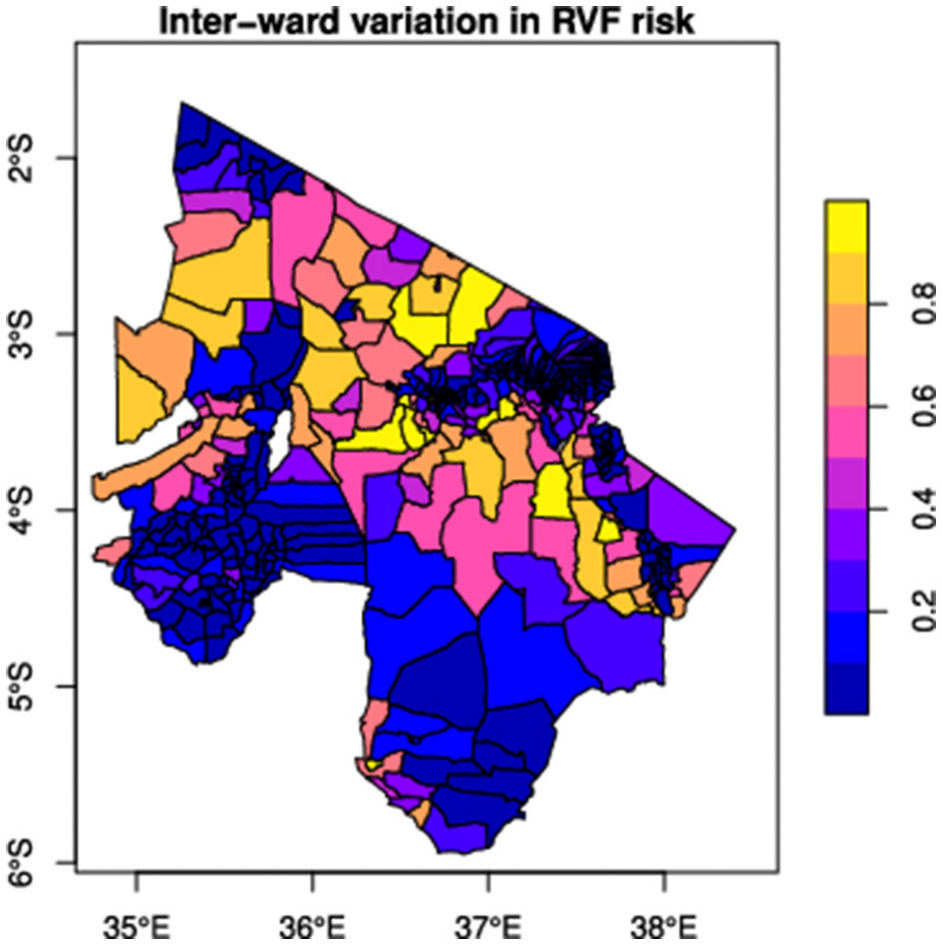
The distribution of RVF emergence risk was simulated by linking disease emergence to climate suitability for RVF vectors. RVF risk score ranges between 0 and 1 (right); a high-risk score represents high RVF risk.

Cattle were moved between network wards through network links every month (30 days) to allow RVF virus to spread between wards. These monthly movements of cattle are processed within the first four days of each month. The network link weights determined the number of cattle moved in the data-driven network. In contrast, cattle movements in the theoretical networks were based on the mean number of animals moved through markets. In each simulation scenario, we select a proportion of nodes for vaccination based on the strategy of interest. Since we are only interested in successful epidemics, we seeded infection in five wards based on disease introduction risk (see Figure 3) and with 10 infected cattle in each of those wards, to reduce the probability of stochastic extinction, as less intensive seeding resulted in many failed simulations. For each ward selected for disease introduction, seeded cases were uniformly distributed between grids of the ward, and the epidemic was allowed to spread locally and through cattle movements within and between wards, respectively. The numbers of animals in each state were updated at daily time steps. All simulations were implemented using the SimInf package (90) within the R statistical software environment (68). SimInf is a flexible framework for data-driven spatiotemporal infectious disease modeling that efficiently handles population demographics and network data (90). It uses continuous-time Markov chains and the Gillespie stochastic simulation algorithm (91), allowing for stochastic transitions between compartments as a Poisson process (90).

### 2.7. Vaccination strategies

To explore the impact of vaccination on RVF virus transmission dynamics, a proportion of nodes (10%, 20%, 30%, 40%, and 50%) were selected for pre-emptive vaccination (i.e. in anticipation of a higher risk of disease outbreaks), at 75% within-node coverage (the proportion of cattle to be vaccinated in each ward to achieve herd immunity, $1-\frac{1}{R_{0}}$). Once vaccinated, each bovid might become immune to the infection with some probability, *e*, which represents the vaccine efficacy. In this model, we assume *e* = 100% (that is, the vaccine is 100% efficacious) and no waning immunity. Immune cattle cannot become infected and do not contribute to disease transmission. The assumption of 100% vaccine efficacy and 75% within-ward coverage can be interpreted as equivalent to 100% coverage with 75% vaccine efficacy.

Given limited resources, we wish to select a proportion of nodes for vaccination to ensure the greatest reduction in epidemic size. Here, we considered four vaccination strategies. These included three network-based strategies, where nodes with high degree, betweenness, or PageRank centrality rank are targeted for vaccination, and a risk-based strategy motivated by habitat suitability for disease occurrence (Figure 3). The aim of comparing network-based against risk-based strategies was to understand whether the risk of introduction of disease is a better determinant of outbreak size than the risks associated with propagation across the network. We also considered random vaccination and a no-vaccination scenario to establish two baseline measures for comparison to targeted vaccination scenarios.

The effectiveness of vaccination strategies under perfect information was measured in terms of percentage reduction in the mean number of wards with at least 0.5% bovid infections (MNWI) under each vaccination scenario relative to the MNWI under the no-vaccination scenario across all 400 simulations. Effectiveness was calculated as (no-vaccination MNWI minus vaccination MNWI)/(no-vaccination MNWI). The threshold of 0.5% bovid infections was chosen to avoid classifying infected wards with very small and short-lived outbreaks, which would be unlikely to be detected in reality (72).

### 2.8. Simulating imperfect network information

To examine the effectiveness of vaccination strategies under conditions of imperfect but unbiased cattle movement network data, we used the following process: (1) given a perfect network *G*(*N, M*), where *N* and *M* are the nodes and link sets, calculate the node-level measure of interest *S*; (2) derive the rank *R*_*S*_ of *S*, and break ties randomly; (3) add normally distributed noise ϵ to the rank *R*_*S*_ such that the actual rank and the noisy rank *R*_*n*_ = rank(*R*_*S*_+ϵ) are correlated by ρ∈(0, 1], where ρ is the Spearman rho rank correlation coefficient and $\epsilon \sim N\left(0,\sigma^{2}\right)$; When σ^2^ = 0 and therefore ρ = 1, the actual rank and the noisy rank are precisely the same (perfect information). As σ^2^ → ∞ and ρ → 0, the noisy rank approaches a random ranking, which does not rely on network information. (4) Select the top-ranked nodes for vaccination based on the noisy rank *R*_*n*_.

We simulated the impact of increasing levels of network data error on the efficacy of network-based targeting of vaccination by investigating a range of ρ values from 1 (perfect network information) to 0 (no network information). Effectiveness was measured in terms of the relative difference in MNWI when 20% of wards are vaccinated, at 75% within ward coverage, using the outcome of random vaccination as a baseline. We also simulated and reported the results for 10%, 30%, 40%, and 50% vaccinated wards in the Supplementary material.

## 3. Results

### 3.1. RVF virus transmission dynamics and the impact of cattle movements

**Figure 4:**
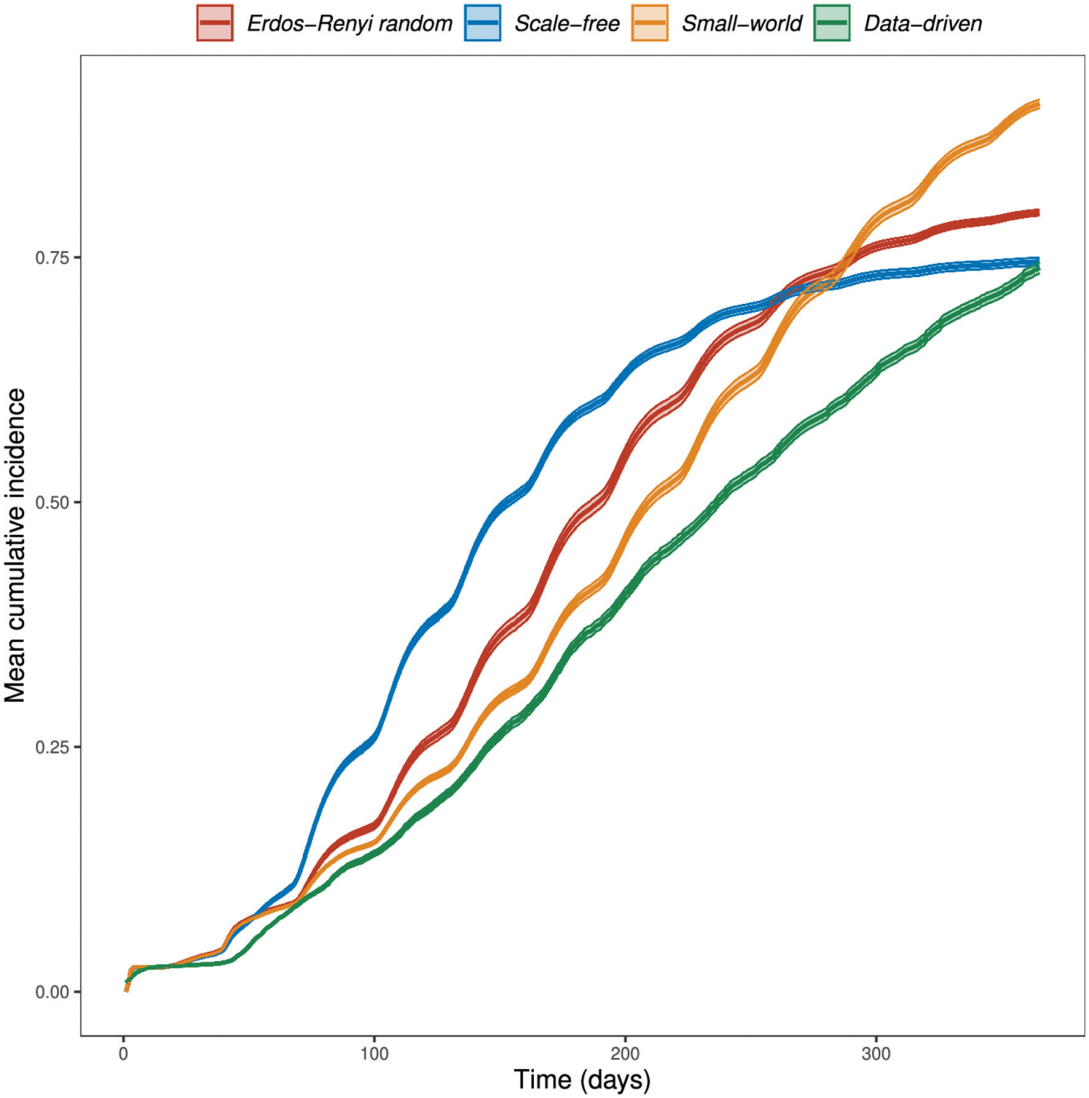
Simulated mean cumulative incidence by network type. The RVF meta-population model was simulated for one year, at a daily time step, with parameter values in Table 2, allowing disease spread within and between populations. Cattle movements between wards occur monthly, with rates determined by the weights of the data-driven network. In theoretical networks, 100 animals were moved between wards every month. This choice was based on the mean number of animals moved through the market network. The plots shown are the mean cumulative incidence of 400 simulations with a 95% confidence interval.

At the end of 365 days in the absence of vaccination, our simulation model resulted in a mean cumulative incidence of infected wards of 80% in ER random networks, 75% in scale-free networks, 91% in small-world networks, and 74% in the data-driven network (Figure 4).

A larger outbreak (proportion of infected wards) was observed more often in small-world networks than in the other two theoretical network types, which can be explained through the size of the giant strongly connected components (GSCC) presented in Table 1. We observed the role of livestock movements on disease spread, occurring every month, through the sudden increase in cumulative incidence following the monthly movements (Figure 4).

### 3.2. Impact of intervention strategies

**Figure 5:**
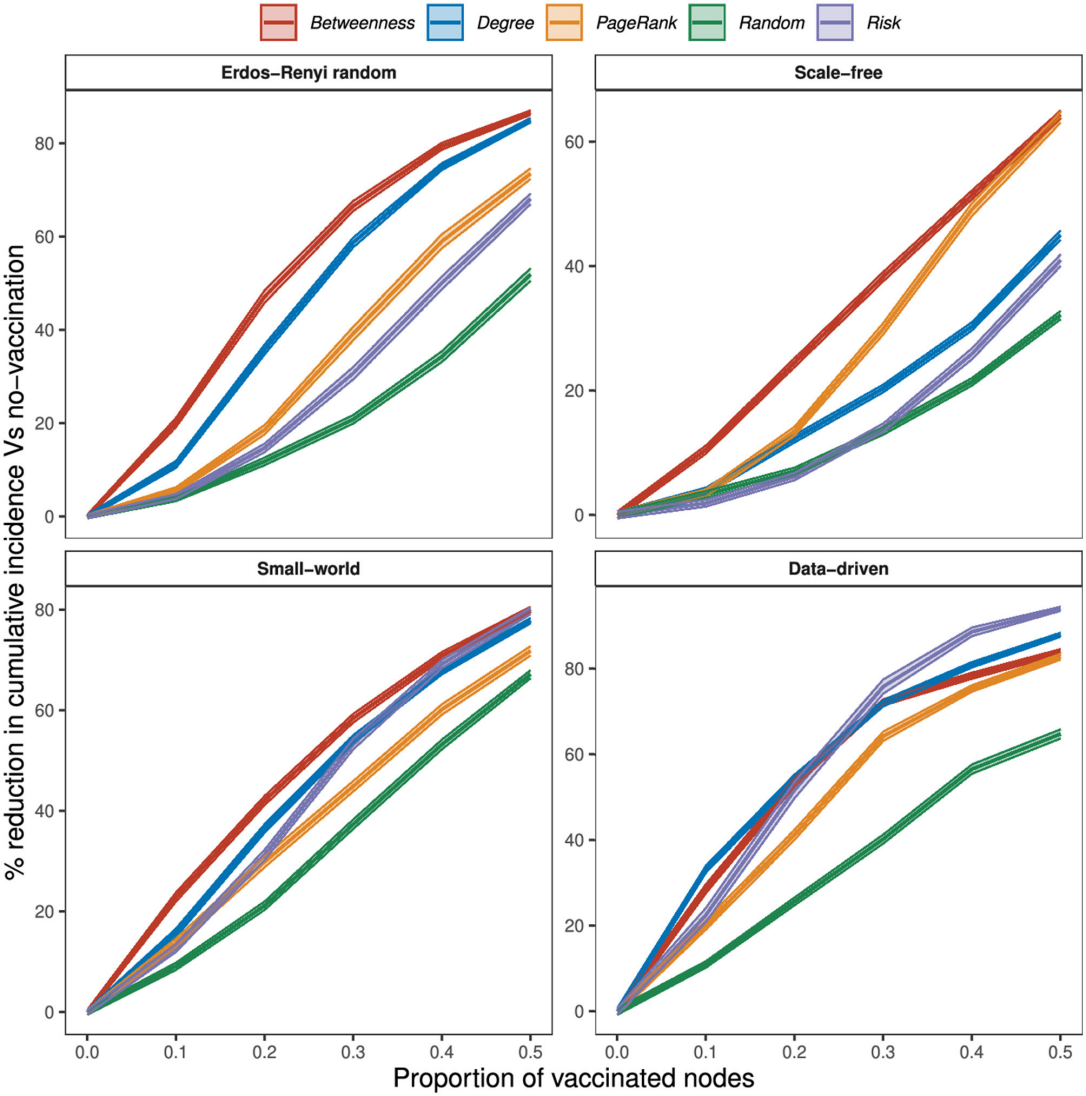
Percentage reduction in node cumulative incidence relative to no vaccination by the proportion of vaccinated nodes, at 75% within-node coverage, for different vaccination strategies.

The effectiveness of targeted strategies varies with network type (Figure 5). Targeting vaccination at 10% of wards that rank highest on degree and betweenness reduced MNWI by about 11 and 20% in ER random networks, 4 and 11% in scale-free networks, 16 and 23% in small-world networks, and 33 and 29% in the data-driven network. When 20% of wards are vaccinated, degree and betweenness strategies reduced MNWI by over 50% in the data-driven network. At 20% degree and betweenness reduced cumulative incidence by about 36 and 47% in ER random networks, 12 and 25% in scale-free networks, 37 and 42% in small-world networks, and 55 and 54% in the data-driven network. Using a PageRank strategy, we need to vaccinate up to 40% of wards to reduce cumulative incidence by 50% in ER random, small-world networks, and scale-free networks; and just over 20% in the data-driven network. When the proportion of vaccinated wards exceeds 20%, vaccination by PageRank shows better performance than degree vaccination in scale-free networks.

In general, random vaccination offers the worst performance in all networks. Risk-based vaccination showed similar effectiveness to the PageRank strategy in all networks when the number of vaccinated wards is not more than 10%, and outperformed the strategy in certain scenarios and networks. Specifically, at 10% of vaccination wards, PageRank and risk vaccination reduced MNWI by only 6 and 4% in ER random networks, 4 and 2% in scale-free networks, 14 and 13% in small-world networks, and 20 and 23% in the data-driven network. In small-world networks, where 30% of vaccination wards were targeted, the risk-based strategy achieved a significant reduction of 53% in MNWI, while PageRank yielded a reduction of 45%. Furthermore, in the data-driven network, vaccination coverage of 20% using the risk-based strategy resulted in an effectiveness of 52%, outperforming PageRank with an effectiveness of 41%.

Our results reveal that vaccination based on the risk of disease emergence is highly effective in the data-driven network. Particularly noteworthy is the superiority of risk-based vaccination over all network-based strategies when the proportion of vaccinated wards exceeds 20%. Conversely, in scenarios where fewer than 20% of wards are vaccinated, degree and betweenness strategies exhibit greater effectiveness. In the data-driven network, all network-based strategies achieve an effectiveness of over 80% when half of the wards are vaccinated. Notably, risk vaccination stands out, achieving an effectiveness of up to 94%.

### 3.3. Effect of adding noise on effectiveness

**Figure 6:**
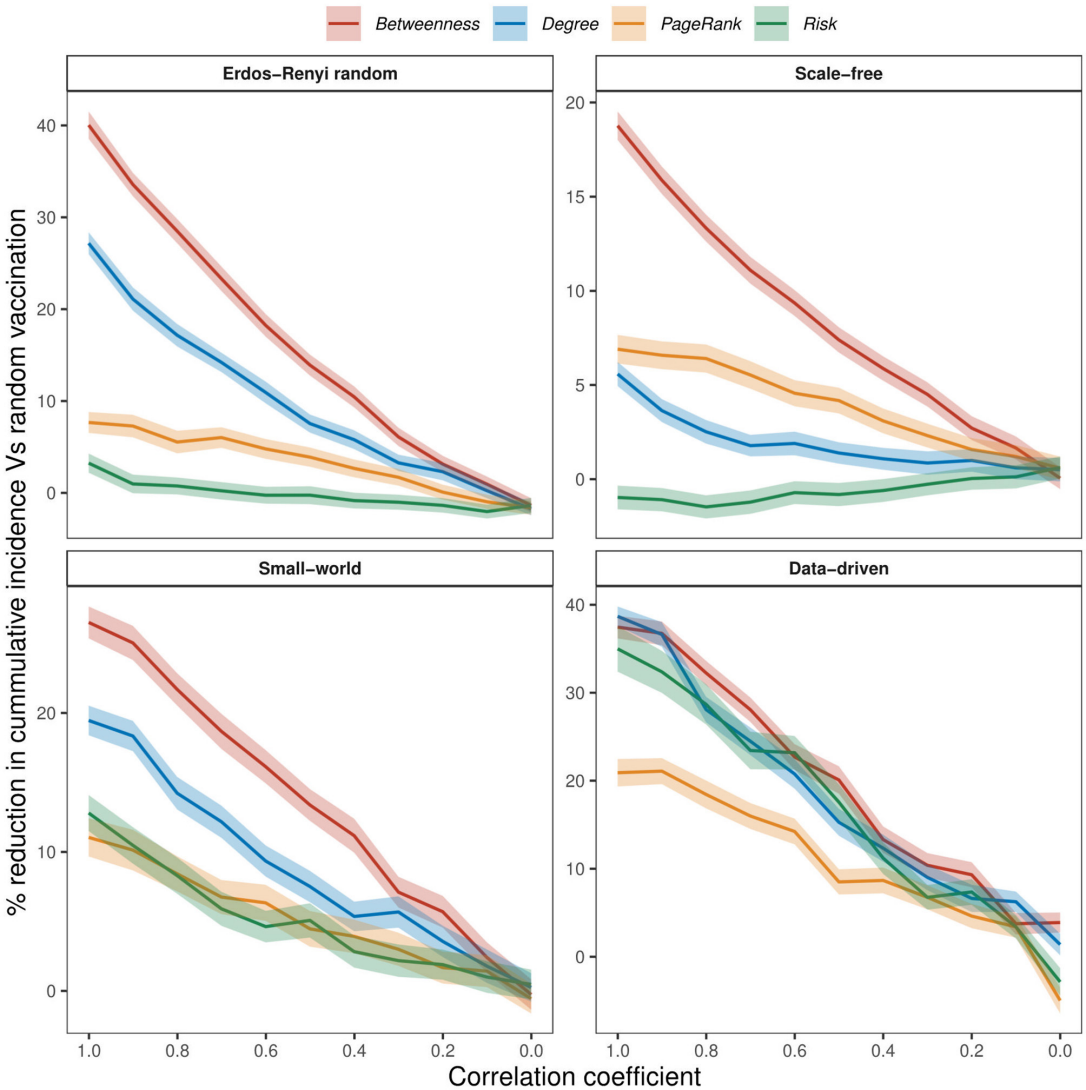
The effect of increasing noise on the effectiveness of vaccination strategies when 20% of nodes are vaccinated, at 75% within-node coverage. Noise was added to simulate conditions of imperfect network information. Perfect and imperfect ranks were compared using Spearman's ρ rank correlation coefficient, with a correlation coefficient of 1 indicating perfect information.

We examined the impact of incomplete network information on the effectiveness of vaccination strategies by introducing noise to our assessment of risks. Figure 6 shows the relative difference in cumulative incidence when 20% of wards are vaccinated using random vaccination as the baseline. Across all networks and for all values of ρ>0, targeted strategies outperformed random vaccination. Even at a very high noise level (low values of ρ), targeted strategies consistently achieved greater reductions in cumulative incidence compared to random vaccination in all networks. One exception is the risk strategy in scale-free networks, where effectiveness fell below that of random vaccination at high ρ values (Figure 6). In general, the effectiveness of targeted strategies decreases almost linearly with increasing noise and converges to random vaccination as ρ approaches 0. Similar trends were observed when 10%, 30%, 40%, and 50% of wards are vaccinated (see Supplementary Figures 2–5).

In the case of perfect network information (ρ = 1), the betweenness vaccination strategy outperformed random vaccination by ~40% in ER random networks, 19% in scale-free networks, 27% in small-world networks, and 37% in the data-driven network (see Figure 6). Both degree and PageRank strategies showed improvements of about 27 and 8% in ER random networks, 6% and 7% in scale-free networks, 19 and 11% in small-world networks, and 39 and 21% in the data-driven network. Similarly, risk vaccination demonstrated better performance than random vaccination in ER random, small-world, and data-driven networks, with improvements of 4%, 13%, and 35%, respectively. However, in scale-free networks, risk vaccination showed a slight decrease of 1% compared to random vaccination.

Both betweenness and degree strategies lost their effectiveness by over 50% in all networks when our knowledge of network information dropped by half (ρ = 0.5). A similar observation was noted about risk strategy in the data-driven and small-world networks, where effectiveness declined by over 50% when our knowledge of disease emergence points is 50% correct. Although the degree and betweenness vaccination strategies outperformed other strategies in the data-driven network at ρ = 1, their effectiveness became comparable to risk-based vaccination when ρ dropped below 0.5.

## 4. Discussion

In this study, we used a meta-population model to simulate the spread of RVF virus on livestock movement networks, examined the impact of network-based and risk-based vaccination strategies, and explored how imperfect information affects their effectiveness. This research addressed a common real-life problem in veterinary research—incomplete and patchy data about livestock movement patterns—arising in Tanzania and many other countries.

The major finding of this investigation is that the loss of effectiveness of targeted vaccination strategies with increasing data imperfection is approximately linear regardless of network structure. Essentially, any improvement in information reliability is equivalently beneficial. These findings are consistent with a similar study conducted by Rosenblatt et al. (56) on immunization strategies in the presence of missing data. Their study also demonstrated that targeted immunization consistently outperforms random immunization, even when confronted with high levels of data imperfection such as missing nodes. Therefore, our results emphasize the robustness and practical significance of targeted vaccination strategies, despite the considerable challenges posed in this case by incomplete network information.

Additional results showed no universally optimal targeting strategy across all network structures and proportions of vaccinated nodes. Nevertheless, vaccination by betweenness and degree strategies are generally the most effective network-based strategies. This observation is robust to very different network structures and corroborates the results of other research studies (31, 40–42, 49, 92). Betweenness-based vaccination emerges as the most effective strategy across all theoretical networks, surpassing other targeted vaccination approaches. Meanwhile, degree-based vaccination demonstrates superior performance among network-based strategies specifically in the data-driven network. Even though the data-driven network is fully connected with a GSCC of 398, the outbreak size was smaller than that of the small-world with a GSCC of 388 network. A possible explanation for this circumstance might be because up to 34% of the link weights in the data-driven network are significantly less than 1, representing lower onward transmission risk through those links.

Under perfect information, we noted a crossover point between the effectiveness of network-based (degree and betweenness) and geographical risk-based vaccination in the data-driven network. For example, when a small proportion of wards are vaccinated (<30%), both degree and betweenness vaccination work better than geographical risk-based vaccination. However, when the proportion of vaccinated wards is higher, risk vaccination outperformed all network-based vaccination. This evidence suggests that if our knowledge of network information were perfect, there might be scenarios where vaccinating the points of RVF virus introduction into the system is preferred over vaccinating the points of onward transmission. In scenarios where we can only cover 20% of wards, our findings show there is a noise level at which the effectiveness of degree and betweenness are no different from the effectiveness of risk vaccination. Again, this observation suggests that if our knowledge of network information is imperfect, depending on how well we know the highly central and high-risk wards, we might choose one strategy over the other when we can only vaccinate a few wards. Because this result is not guaranteed to be robust to changing assumptions of the underlying network structures, we are unable to extrapolate it to all networks. For instance, in random and scale-free networks, all targeting strategies are perfectly linear with increasing noise and there is no crossover.

There are some potential limitations of this study. We have shown that the strategy of targeting using network data is effective and robust to data errors to some degree. However, the incorporation of finer-scale data would be required to make the model applicable to predicting the effectiveness of targeting specific wards. The network used in this study is generated based on the outcome of Chaters et al. (31), and it is not an exact representation of the Tanzanian cattle movement system. The data used in the study by Chaters et al. (31) relied on fragmented and patchy paper-based market permit movement data, which may limit the accuracy of the inferred network. Additionally, the network does not capture the complexities of non-market movements, such as movements to resource locations and private sales. In our model, we assumed that cattle movements between wards happen monthly and are processed as scheduled events within the first four days of every month. Subsequently, we assumed no further movements between wards until the next month. This pattern of movements, though motivated by the analysis of Chaters et al. (31), may have important implications for the spread of RVF across wards. However, the flexibility of our model framework allows for the incorporation of regular movement patterns, say, weekly and fortnightly markets. The number of nodes used to generate theoretical networks was kept very small to mimic the data-driven system, which might affect our ability to generalize results to larger networks. The size of a network can influence its overall structure and the interactions between its nodes. As the number of nodes in a network increases, the number of possible connections between nodes also increases, which can result in more intricate network structures. This, in turn, can impact the dynamics of disease transmission within the network. It is worth noting that although this study investigates the impact of imperfect information, we did not address the impact of information bias, which may present a more challenging problem to overcome. In addition, we did not evaluate the impact of the bias and error associated with noise in the risk of RVF, as expressed through NDVI. Whilst NDVI can be accurately measured, our strategy of targeting introduction into the highest NDVI wards may have relevant inaccuracies due to the complexity of the correlation between NDVI and the emergence of RVF at the ward scale (82). Furthermore, we assumed perfect vaccine efficacy and no loss of vaccine-induced immunity. However, studies have shown that vaccine efficacy varies depending on the type of vaccine and the livestock species (27, 28). For instance, Njenga et al. (29) reported that the RVF virus Clone 13 vaccine showed greater effectiveness in small ruminants than in cattle, and it elicited a long-lasting antibody response that lasted up to 12 months (93). Considering the timing of preemptive vaccination in cattle, there is a potential for immunity to wane before the end of the simulation period. The combined effect of imperfect vaccine protection and potential waning immunity could impact the effectiveness of vaccination strategies. Several studies have found that mixing livestock herds with wildlife increases RFV virus seroprevalence (94–96). However, we did not investigate how wildlife might impact intervention scenarios.

Lastly, our model did not consider mosquito-vector dynamics. This decision was due to insufficient data about the abundance and activities of specific mosquitoes (*Aedes* species) capable of transmitting the RVF virus. It is evident that mosquitoes contribute to keeping the RVF virus within the enzootic cycle by transmitting the virus from host to host, leading to the persistence of RVF. To provide a more comprehensive understanding of RVF dynamics, future studies could extend our model to explicitly incorporate mosquito population dynamics and consider multiple hosts where relevant data is available. We recommend exploring the inclusion of additional complexities, such as heterogeneity in host susceptibility (e.g., age structure), finer-scale livestock movement systems (e.g., at the village level) and diverse intervention strategies (e.g., movement bans). We used NDVI as an example of how emergence and transmission risk might vary over space, but a potential improvement to the model would be to link RVF risk to a wider range of dynamic habitat predictors. Additionally, investigating the role of non-market movements on network structures would provide valuable insights. By investigating these aspects, we can enhance our understanding of the dynamics of RVF and other similar zoonotic diseases. This knowledge will contribute to the development of more effective strategies for the control and prevention of these diseases.

Our study provides a framework to examine the hypothesis that targeted strategies, which have been extensively studied and proven highly effective under perfect information, can still be beneficial even under imperfect information. Our results suggest that targeting interventions, even with limited information, is likely to reduce the spread of infectious livestock diseases. Furthermore, our findings highlight the significant gains that can be achieved by improving information and data availability for livestock movement. In light of these findings, it is crucial to emphasize the importance of striving for comprehensive and high-quality data collection. Therefore, we strongly recommend prioritizing robust data collection practices whenever feasible. By improving data quality and carefully designing and planning network-driven intervention strategies, particularly in regions like sub-Saharan Africa where livestock movement data is often incomplete, we can enhance disease control efforts and mitigate the impact of infectious diseases on livestock populations.

## Data availability statement

The data analyzed in this study is subject to the following licenses/restrictions: the dataset used for this analysis records livestock movements in northern Tanzania recorded from 30,000 Livestock Movement Permits. This dataset belongs to the Ministry of Livestock and Fisheries of Tanzania, and we do not have permission to share it publicly. This also applies to the data on the number of cattle in each ward. Requests to access these datasets should be directed to: PJ, paul.johnson@glasgow.ac.uk.

## Author contributions

TS, PJ, JE, SC, and RK conceptualized and formulated the model. TS wrote the simulation code, analyzed the model, produced visualizations, interpreted results, and wrote the original draft of the manuscript. All authors reviewed the manuscript and approved the submitted version.

## References

[1] Robinson TP Thornton PK Franceschini G Kruska RL Chiozza F Notenbaert AMO. Global Livestock Production Systems. Rome: Food and Agriculture Organization of the United Nations (FAO) and International Livestock Research Institute (ILRI) (2011), p. 152.

[2] Katia C Nsiima L Zezza A. Livestock and Livelihoods in Rural Tanzania. Rome: World Bank, FAO, AU-IBAR, ILRI and the Tanzania Ministry of Livestock and Fisheries Development (2012).

[3] Perry B Sones K. Poverty reduction through animal health. Science. (2007) 315:333–4. 10.1126/science.1138614 17234933

[4] Gebreyes WA Dupouy-Camet J Newport MJ Oliveira CJ Schlesinger LS Saif YM. The global one health paradigm: challenges and opportunities for tackling infectious diseases at the human, animal, and environment interface in low-resource settings. PLoS Negl Trop Dis. (2014) 8:e3257. 10.1371/journal.pntd.0003257 25393303 PMC4230840

[5] Grace D Mutua F Ochungo P Kruska R Jones K Brierley L. Mapping of Poverty and Likely Zoonoses Hotspot. Nairobi: International Livestock Research Institute (2012).

[6] Gibb R Redding DW Chin KQ Donnelly CA Blackburn TM Newbold T. Zoonotic host diversity increases in human-dominated ecosystems. Nature. (2020) 584:398–402. 10.1038/s41586-020-2562-8 32759999

[7] Omambia CS Gu Y. The cost of climate change in Tanzania: impacts and adaptations. J Am Sci. (2010) 6:182–96.

[8] Thornton PK. Livestock production: recent trends, future prospects. Philos Trans R Soc B Biol Sci. (2010) 365:2853–67. 10.1098/rstb.2010.0134 20713389 PMC2935116

[9] Keesing F Belden LK Daszak P Dobson A Harvell CD Holt RD. Impacts of biodiversity on the emergence and transmission of infectious diseases. Nature. (2010) 468:647–52. 10.1038/nature09575 21124449 PMC7094913

[10] Kao RR Danon L Green DM Kiss IZ. Demographic structure and pathogen dynamics on the network of livestock movements in Great Britain. Proc R Soc B Biol Sci. (2006) 273:1999–2007. 10.1098/rspb.2006.3505 16846906 PMC1635475

[11] Gibbens J Wilesmith J Sharpe C Mansley L Michalopoulou E Ryan J. Descriptive epidemiology of the 2001 foot-and-mouth disease epidemic in Great Britain: the first five months. Vet Rec. (2001) 149:729–43. 10.1136/vr.149.24.729 11808655

[12] Kao RR. The role of mathematical modelling in the control of the 2001 FMD epidemic in the UK. Trends Microbiol. (2002) 10:279–86. 10.1016/S0966-842X(02)02371-5 12088664

[13] Anderson RM May RM. Infectious Diseases of Humans: Dynamics and Control. Oxford: Oxford University Press (1992).

[14] Keeling MJ Rohani P. Modeling Infectious Diseases in Humans and Animals. Princeton, NJ: Princeton University Press. (2011). 10.2307/j.ctvcm4gk0

[15] Ball F Mollison D Scalia-Tomba G. Epidemics with two levels of mixing. Ann Appl Probab. (1997) 7:46–89. 10.1214/aoap/1034625252

[16] Bjørnstad ON Finkenstädt BF Grenfell BT. Dynamics of measles epidemics: estimating scaling of transmission rates using a time series SIR model. Ecol Monogr. (2002) 72:169–84. 10.1890/0012-9615(2002)072[0169:DOMEES]2.0.CO;2

[17] Lloyd-Smith JO Schreiber SJ Kopp PE Getz WM. Superspreading and the effect of individual variation on disease emergence. Nature. (2005) 438:355–9. 10.1038/nature04153 16292310 PMC7094981

[18] Bansal S Grenfell BT Meyers LA. When individual behaviour matters: homogeneous and network models in epidemiology. J R Soc Interface. (2007) 4:879–91. 10.1098/rsif.2007.1100 17640863 PMC2394553

[19] Kiss IZ Miller JS Simon P. Mathematics of Epidemics on Networks: From Exact to Approximate Models. New York, NY: Springer International Publishing (2017). 10.1007/978-3-319-50806-1

[20] Centers for Disease Control Prevention. One Health zoonotic disease prioritization for multisectoral engagement in Tanzania. Centers for Disease Control and Prevention (2017). Available online at: https://www.cdc.gov/onehealth/pdfs/tanzania-report-508.pdf (accessed March 05, 2022).

[21] Mpolya EA Lembo T Lushasi K Mancy R Mbunda EM Makungu S. Toward elimination of dog-mediated human rabies: experiences from implementing a large-scale demonstration project in southern Tanzania. Front Vet Sci. (2017) 4:21. 10.3389/fvets.2017.00021 28321400 PMC5337520

[22] Sindato C Karimuribo E Mboera LE. The epidemiology and socio-economic impact of Rift Valley fever in Tanzania: a review. Tanzan J Health Res. (2011) 13:305–18. 10.4314/thrb.v13i5.1 26591986

[23] The New Humanitarian. Tanzania: Rift Valley Fever Under Control, Says Government (2021). Available online at: https://reliefweb.int/report/united-republic-tanzania/tanzania-rift-valley-fever-under-control-says-government (accessed May 21, 2023).

[24] Sindato C Karimuribo ED Swai ES Mboera LE Rweyemamu MM Paweska JT. Safety, immunogenicity and antibody persistence of rift valley fever virus clone 13 vaccine in sheep, goats and cattle in Tanzania. Front Vet Sci. (2021) 8:779858. 10.3389/fvets.2021.779858 34977212 PMC8718550

[25] Smithburn K. Rift Valley fever: the neurotropic adaptation of the virus and the experimental use of this modified virus as a vaccine. Br J Exp Pathol. (1949) 30:1. 18128091 PMC2073103

[26] Botros B Omar A Elian K Mohamed G Soliman A Salib A. Adverse response of non-indigenous cattle of European breeds to live attenuated Smithburn Rift Valley fever vaccine. J Med Virol. (2006) 78:787–91. 10.1002/jmv.20624 16628582

[27] Kitandwe PK McKay PF Kaleebu P Shattock RJ. An overview of rift valley fever vaccine development strategies. Vaccines. (2022) 10:1794. 10.3390/vaccines10111794 36366303 PMC9697312

[28] Faburay B LaBeaud AD McVey DS Wilson WC Richt JA. Current status of Rift Valley fever vaccine development. Vaccines. (2017) 5:29. 10.3390/vaccines5030029 28925970 PMC5620560

[29] Njenga MK Njagi L Thumbi SM Kahariri S Githinji J Omondi E. Randomized controlled field trial to assess the immunogenicity and safety of Rift Valley fever Clone 13 vaccine in livestock. PLoS Negl Trop Dis. (2015) 9:e0003550. 10.1371/journal.pntd.0003550 25756501 PMC4355591

[30] de Glanville WA Davis A Allan KJ Buza J Claxton JR Crump JA. Classification and characterisation of livestock production systems in northern Tanzania. PLoS ONE. (2020) 15:e0229478. 10.1371/journal.pone.0229478 33378382 PMC7773236

[31] Chaters GL Johnson PCD Cleaveland S Crispell J De Glanville WA Doherty T. Analysing livestock network data for infectious disease control: an argument for routine data collection in emerging economies. Philos Trans R Soc B Biol Sci. (2019) 374:20180264. 10.1098/rstb.2018.0264 31104601 PMC6558568

[32] Hanski I. Metapopulation dynamics. Nature. (1998) 396:41–9. 10.1038/23876

[33] Hess G. Disease in metapopulation models: implications for conservation. Ecology. (1996) 77:1617–32. 10.2307/226555620715611

[34] Pastor-Satorras R Vespignani A. Immunization of complex networks. Phys Rev E. (2002) 65:036104. 10.1103/PhysRevE.65.036104 11909162

[35] Wang Z Bauch CT Bhattacharyya S d'Onofrio A Manfredi P Perc M. Statistical physics of vaccination. Phys Rep. (2016) 664:1–113. 10.1016/j.physrep.2016.10.006

[36] Cohen R Havlin S Ben-Avraham D. Efficient immunization strategies for computer networks and populations. Phys Rev Lett. (2003) 91:247901. 10.1103/PhysRevLett.91.247901 14683159

[37] Holme P. Efficient local strategies for vaccination and network attack. Europhys Lett. (2004) 68:908. 10.1209/epl/i2004-10286-2

[38] Albert R Barabási AL. Statistical mechanics of complex networks. Rev Mod Phy. (2002) 74:47–97. 10.1103/RevModPhys.74.47

[39] Kiss IZ Green DM Kao RR. Infectious disease control using contact tracing in random and scale-free networks. J R Soc Interface. (2006) 3:55–62. 10.1098/rsif.2005.0079 16849217 PMC1618487

[40] Hébert-Dufresne L Allard A Young JG Dubé LJ. Global efficiency of local immunization on complex networks. Sci Rep. (2013) 3:1–8. 10.1038/srep02171 23842121 PMC3707349

[41] Gates MC Woolhouse MEJ. Controlling infectious disease through the targeted manipulation of contact network structure. Epidemics. (2015) 12:11–9. 10.1016/j.epidem.2015.02.008 26342238 PMC4728197

[42] Keeling M Woolhouse M May R Davies G Grenfell BT. Modelling vaccination strategies against foot-and-mouth disease. Nature. (2003) 421:136–42. 10.1038/nature01343 12508120

[43] Kiss IZ Green DM Kao RR. The network of sheep movements within Great Britain: network properties and their implications for infectious disease spread. J R Soc Interface. (2006) 3:669–77. 10.1098/rsif.2006.0129 16971335 PMC1664651

[44] Machado G Galvis JA Lopes FPN Voges J Medeiros AAR Cárdenas NC. Quantifying the dynamics of pig movements improves targeted disease surveillance and control plans. Transbound Emerg Dis. (2021) 68:1663–75. 10.1111/tbed.13841 32965771

[45] Roy S McElwain TF Wan Y. A network control theory approach to modeling and optimal control of zoonoses: case study of brucellosis transmission in sub-Saharan Africa. PLoS Negl Trop Dis. (2011) 5:e1259. 10.1371/journal.pntd.0001259 22022621 PMC3191122

[46] Motta P Porphyre T Handel I Hamman SM Ngu Ngwa V Tanya V. Implications of the cattle trade network in Cameroon for regional disease prevention and control. Sci Rep. (2017) 7:1–13. 10.1038/srep43932 28266589 PMC5339720

[47] Marquetoux N Stevenson MA Wilson P Ridler A Heuer C. Using social network analysis to inform disease control interventions. Prev Vet Med. (2016) 126:94–104. 10.1016/j.prevetmed.2016.01.022 26883965

[48] Laager M Mbilo C Madaye EA Naminou A Léchenne M Tschopp A. The importance of dog population contact network structures in rabies transmission. PLoS Negl Trop Dis. (2018) 12:e0006680. 10.1371/journal.pntd.0006680 30067733 PMC6089439

[49] Eames KT Read JM Edmunds WJ. Epidemic prediction and control in weighted networks. Epidemics. (2009) 1:70–6. 10.1016/j.epidem.2008.12.001 21352752

[50] Craft ME. Infectious disease transmission and contact networks in wildlife and livestock. Philos Trans R Soc B Biol Sci. (2015) 370:20140107. 10.1098/rstb.2014.0107 25870393 PMC4410373

[51] Borgatti SP Carley KM Krackhardt D. On the robustness of centrality measures under conditions of imperfect data. Soc Netw. (2006) 28:124–36. 10.1016/j.socnet.2005.05.001

[52] Wang DJ Shi X McFarland DA Leskovec J. Measurement error in network data: a re-classification. Soc Netw. (2012) 34:396–409. 10.1016/j.socnet.2012.01.003

[53] Galaskiewicz J. Estimating point centrality using different network sampling techniques. Soc Netw. (1991) 13:347–86. 10.1016/0378-8733(91)90002-B

[54] Carley KM Lee JS Krackhardt D. Destabilizing networks. Connections. (2002) 24:79–92.

[55] Costenbader E Valente TW. The stability of centrality measures when networks are sampled. Soc Netw. (2003) 25:283–307. 10.1016/S0378-8733(03)00012-1

[56] Rosenblatt SF Smith JA Gauthier GR Hébert-Dufresne L. Immunization strategies in networks with missing data. PLoS Comput Biol. (2020) 16:e1007897. 10.1371/journal.pcbi.1007897 32645081 PMC7386582

[57] Yang Y McKhann A Chen S Harling G Onnela JP. Efficient vaccination strategies for epidemic control using network information. Epidemics. (2019) 27:115–22. 10.1016/j.epidem.2019.03.002 30878314 PMC6677279

[58] National National Bureau of Statistics URT. 2012 Population and Housing Census: Tanzania Regional Profiles. Dar es Salaam: Ministry of Finance (2016).

[59] Ekwem D Morrison TA Reeve R Enright J Buza J Shirima G. Livestock movement informs the risk of disease spread in traditional production systems in East Africa. Sci Rep. (2021) 11:1–13. 10.1038/s41598-021-95706-z 34385539 PMC8361167

[60] Chaters G. An Evaluation of the Influence of Livestock Movements on the Transmission, Spread and Persistence of Infectious Diseases in Northern Tanzania. Glasgow: University of Glasgow (2021).

[61] Pica-Ciamarra U, Baker, D, Chassama, J, Fadiga, M, Nsiima, L,. Linking Smallholders to Livestock Markets in Tanzania: Combining Market Household Survey Data. The World Bank Group (2011). Available online at: https://openknowledge.worldbank.org/server/api/core/bitstreams/a0046cc7-b0f0-53b5-986a-0e0991b52038/content (accessed September 18, 2022).

[62] Kahle D Wickham H. ggmap: spatial visualization with ggplot2. R J. (2013) 5:144–61. 10.32614/RJ-2013-014

[63] Bollobás B Béla B. Random graphs. 73. Cambridge, MA: Cambridge University Press (2001).

[64] Watts DJ Strogatz SH. Collective dynamics of “small-world” networks. Nature. (1998) 393:440–2. 10.1038/30918 9623998

[65] Saramäki J Kaski K. Modelling development of epidemics with dynamic small-world networks. J Theor Biol. (2005) 234:413–421. 10.1016/j.jtbi.2004.12.003 15784275

[66] Erdős P Rényi A. On the evolution of random graphs. Publ Math Inst Hungarian Acad Sci. (1960) 5:17-61.

[67] Csardi G Nepusz T. The igraph software package for complex network research. Int J Complex Syst. (2006) 1695:1–9.

[68] R Core Team. R: A Language Environment for Statistical Computing. Vienna (2021). Available online at: https://www.R-project.org/ (accessed September 18, 2022).

[69] Freeman LC. Centrality in social networks conceptual clarification. Soc Netw. (1978) 1:215–39. 10.1016/0378-8733(78)90021-7

[70] Stein RA. Super-spreaders in infectious diseases. Int J Infect Dis. (2011) 15:e510–3. 10.1016/j.ijid.2010.06.020 21737332 PMC7110524

[71] Keeling MJ Eames KT. Networks and epidemic models. J R Soc Interface. (2005) 2:295–307. 10.1098/rsif.2005.0051 16849187 PMC1578276

[72] Salathé M Jones JH. Dynamics and control of diseases in networks with community structure. PLoS Comput Biol. (2010) 6:e1000736. 10.1371/journal.pcbi.1000736 20386735 PMC2851561

[73] Ghoshal G Barabási AL. Ranking stability and super-stable nodes in complex networks. Nat Commun. (2011) 2:1–7. 10.1038/ncomms1396 21772265

[74] Miller JC Hyman JM. Effective vaccination strategies for realistic social networks. Phys A: Stat Mech Appl. (2007) 386:780–5. 10.1016/j.physa.2007.08.05424787718

[75] Pastor-Satorras R Vespignani A. Epidemic spreading in scale-free networks. Phys Rev Lett. (2001) 86:3200–3. 10.1103/PhysRevLett.86.3200 11290142

[76] Girvan M Newman ME. Community structure in social and biological networks. Proc Nat Acad Sci. (2002) 99:7821–6. 10.1073/pnas.122653799 12060727 PMC122977

[77] Page L Brin S Motwani R Winograd T. The PageRank Citation Ranking: Bringing Order to the Web. Stanford, CA: Stanford InfoLab. (1999).

[78] Bird BH Ksiazek TG Nichol ST MacLachlan NJ. Rift Valley fever virus. J Am Vet Med Assoc. (2009) 234:883–93. 10.2460/javma.234.7.883 19335238

[79] Gubler DJ Reiter P Ebi KL Yap W Nasci R Patz JA. Climate variability and change in the United States: potential impacts on vector-and rodent-borne diseases. Environ Health Perspect. (2001) 109:223–33. 10.1289/ehp.109-1240669 11359689 PMC1240669

[80] Kilpatrick AM Randolph SE. Drivers, dynamics, and control of emerging vector-borne zoonotic diseases. Lancet. (2012) 380:1946–55. 10.1016/S0140-6736(12)61151-9 23200503 PMC3739480

[81] Ahmed KMS Hamid AA Doka A. Investigation of spatial risk factors for RVF disease occurrence using remote sensing & GIS a case study: Sinnar State, Sudan. J Geogr Inf Syst. (2015) 7:226. 10.4236/jgis.2015.72019

[82] Anyamba A Chretien JP Small J Tucker CJ Formenty PB Richardson JH. Prediction of a Rift Valley fever outbreak. Proc Nat Acad Sci. (2009) 106:955–9. 10.1073/pnas.0806490106 19144928 PMC2626607

[83] Linthicum KJ Anyamba A Tucker CJ Kelley PW Myers MF Peters CJ. Climate and satellite indicators to forecast Rift Valley fever epidemics in Kenya. Science. (1999) 285:397–400. 10.1126/science.285.5426.397 10411500

[84] Métras R Fournié G Dommergues L Camacho A Cavalerie L Mérot P. Drivers for Rift Valley fever emergence in Mayotte: a Bayesian modelling approach. PLoS Neglect Trop Dis. (2017) 11:e0005767. 10.1371/journal.pntd.0005767 28732006 PMC5540619

[85] Gaff HD Hartley DM Leahy NP. An epidemiological model of Rift Valley fever. Electron J Differ Equ. (2007) 2007:1-12.22924058

[86] Mpeshe SC Luboobi LS Nkansah-Gyekye Y. Modeling the impact of climate change on the dynamics of Rift Valley fever. Comput Math Methods Med. (2014) 2014:1–12. 10.1155/2014/627586 24795775 PMC3985190

[87] Braks M Mancini G de Swart M Goffredo M. Risk of vector-borne diseases for the EU: entomological aspects: part 2. EFSA Support Publ. (2017) 14:1184e. 10.2903/sp.efsa.2017.EN-1184

[88] Dungu B Louw I Lubisi A Hunter P von Teichman BF Bouloy M. Evaluation of the efficacy and safety of the Rift Valley Fever Clone 13 vaccine in sheep. Vaccine. (2010) 28:4581–7. 10.1016/j.vaccine.2010.04.085 20470792

[89] von Teichman B Engelbrecht A Zulu G Dungu B Pardini A Bouloy M. Safety and efficacy of Rift Valley fever Smithburn and Clone 13 vaccines in calves. Vaccine. (2011) 29:5771–7. 10.1016/j.vaccine.2011.05.055 21664400

[90] Widgren S Bauer P Eriksson R Engblom S. SimInf: an R package for data-driven stochastic disease spread simulations. J Stat Softw. (2019) 91:1–42. 10.18637/jss.v091.i12

[91] Gillespie DT. Exact stochastic simulation of coupled chemical reactions. J Phys Chem. (1977) 81:2340–61. 10.1021/j100540a00819691379

[92] Shams B. Using network properties to evaluate targeted immunization algorithms. Netw Biol. (2014) 4:74.

[93] Daouam S Ghzal F Naouli Y Tadlaoui K Ennaji M Oura C. Safety and immunogenecity of a live attenuated Rift Valley fever vaccine (CL13T) in camels. BMC Vet Res. (2016) 12:1–5. 10.1186/s12917-016-0775-8 27457539 PMC4960673

[94] Atuman Y Kudi C Abdu P Okubanjo O Wungak Y Ularamu H. Serological evidence of antibodies to Rift Valley fever virus in wild and domestic animals in Bauchi State, Nigeria. Vet Med Int. (2022) 2022, 1–7. 10.1155/2022/6559193 35340539 PMC8942677

[95] Daszak P Cunningham AA Hyatt AD. Emerging infectious diseases of wildlife-threats to biodiversity and human health. Science. (2000) 287:443–9. 10.1126/science.287.5452.443 10642539

[96] Rostal MK Liang JE Zimmermann D Bengis R Paweska J Karesh WB. Rift Valley fever: does wildlife play a role? ILAR J. (2017) 58:359–70. 10.1093/ilar/ilx023 28985319

