# Supplementary material for: Modeling the effectiveness of targeting Rift Valley fever virus vaccination using imperfect network information
Source: Front Vet Sci. 2023 Jun 29;10:1049633. doi: 10.3389/fvets.2023.1049633 (PMC10340087; doi:10.3389/fvets.2023.1049633)
Supplement: Supplementary file 1 [file Data_Sheet_1.PDF]

Supplementary Material

1 SUPPLEMENTARY FIGURES

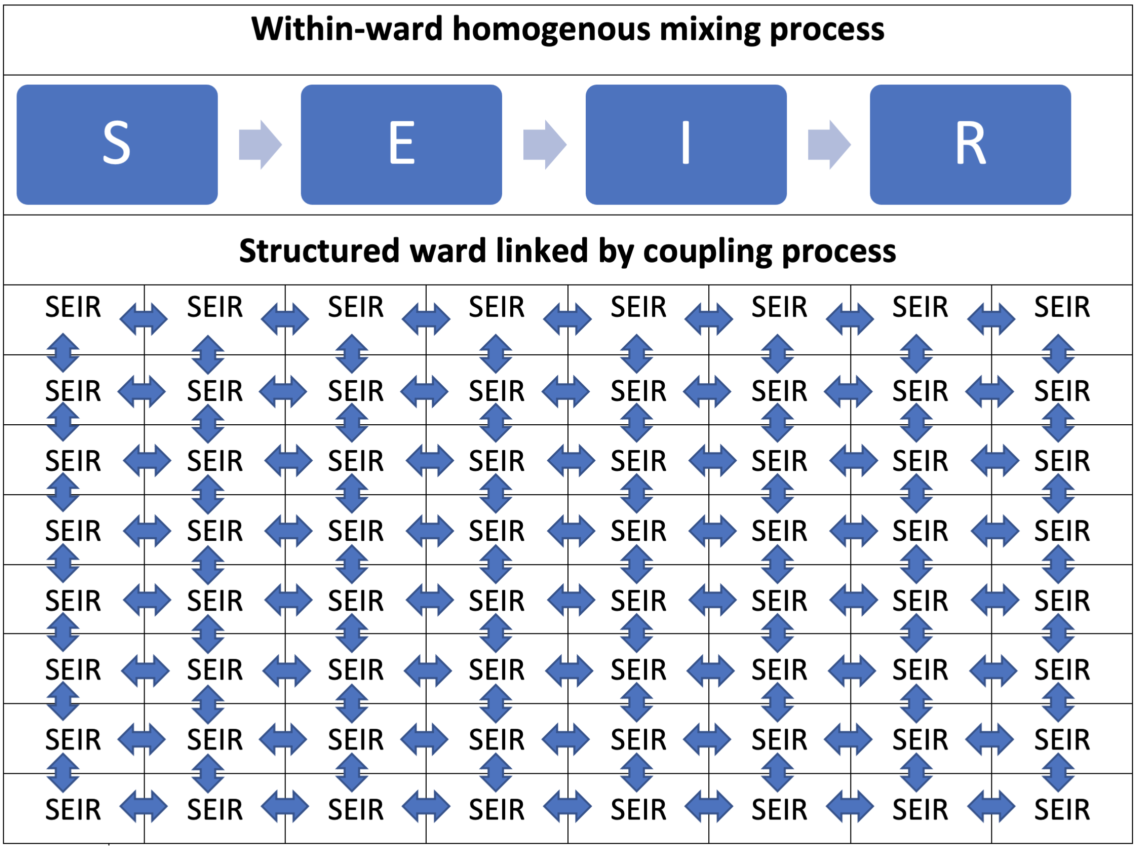

Figure S1. Structured SEIR model describing the transmission of Rift Valley fever virus within-wards in northern Tanzania. Cattle movement data were available describing cattle movements between administrative wards in northern Tanzania and this is the fundamental unit of our data-driven network model. Each ward was divided into 8×8 gird cells to simulate local within-ward transmission driven by spatial transmission. A homogeneous mixing SEIR model was used to describe the transmission dynamics of RVF virus within each cell. Transmission between adjacent cells was allowed through spatial coupling. The grey arrows represent the flow of cattle between four states disease states–susceptibles (S), exposed (E), infectious (I), and recovered (R).

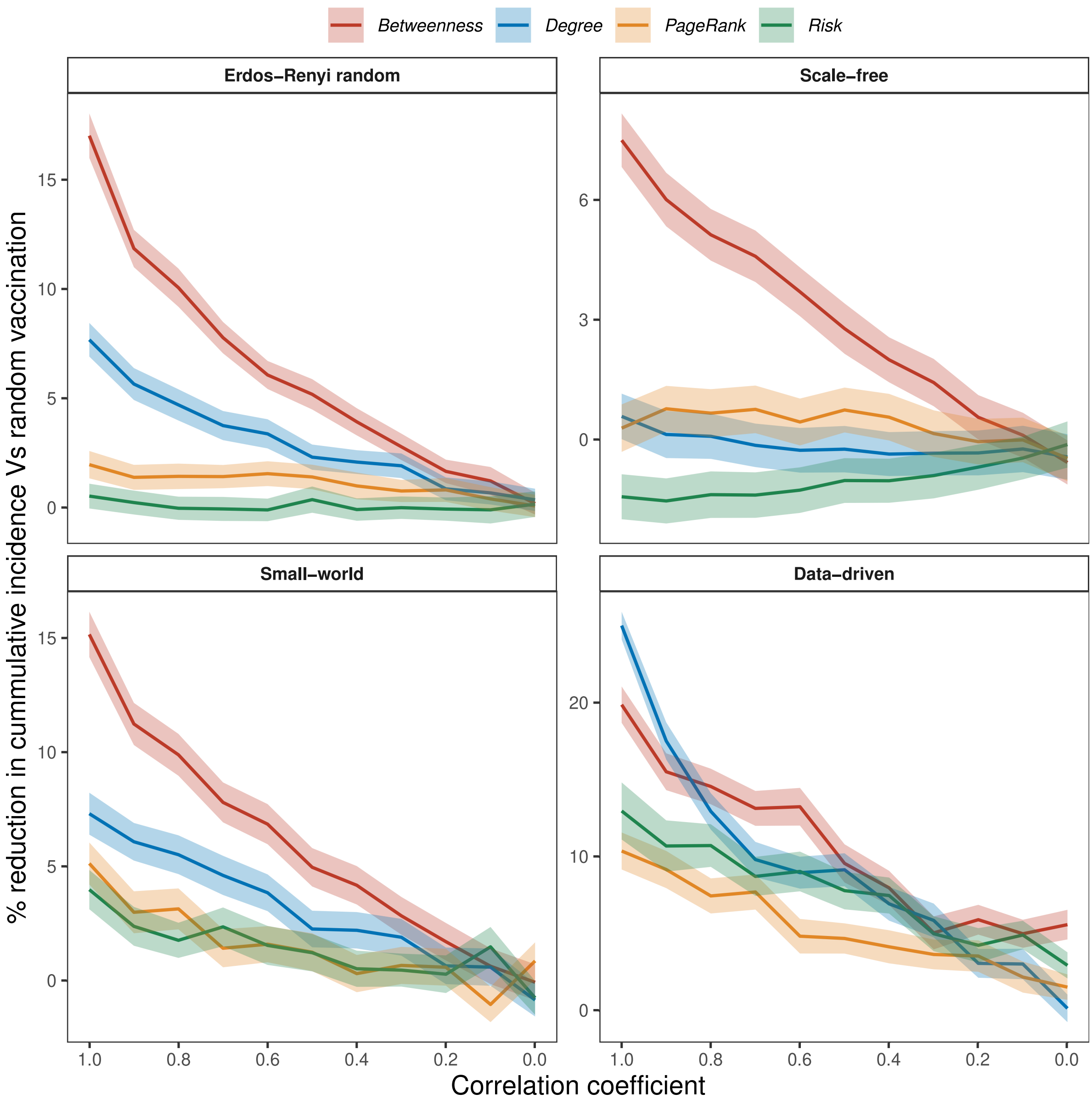

Figure S2. The effect of increasing noise on the effectiveness of vaccination strategies when 10% of nodes are vaccinated, at 75% within-node coverage

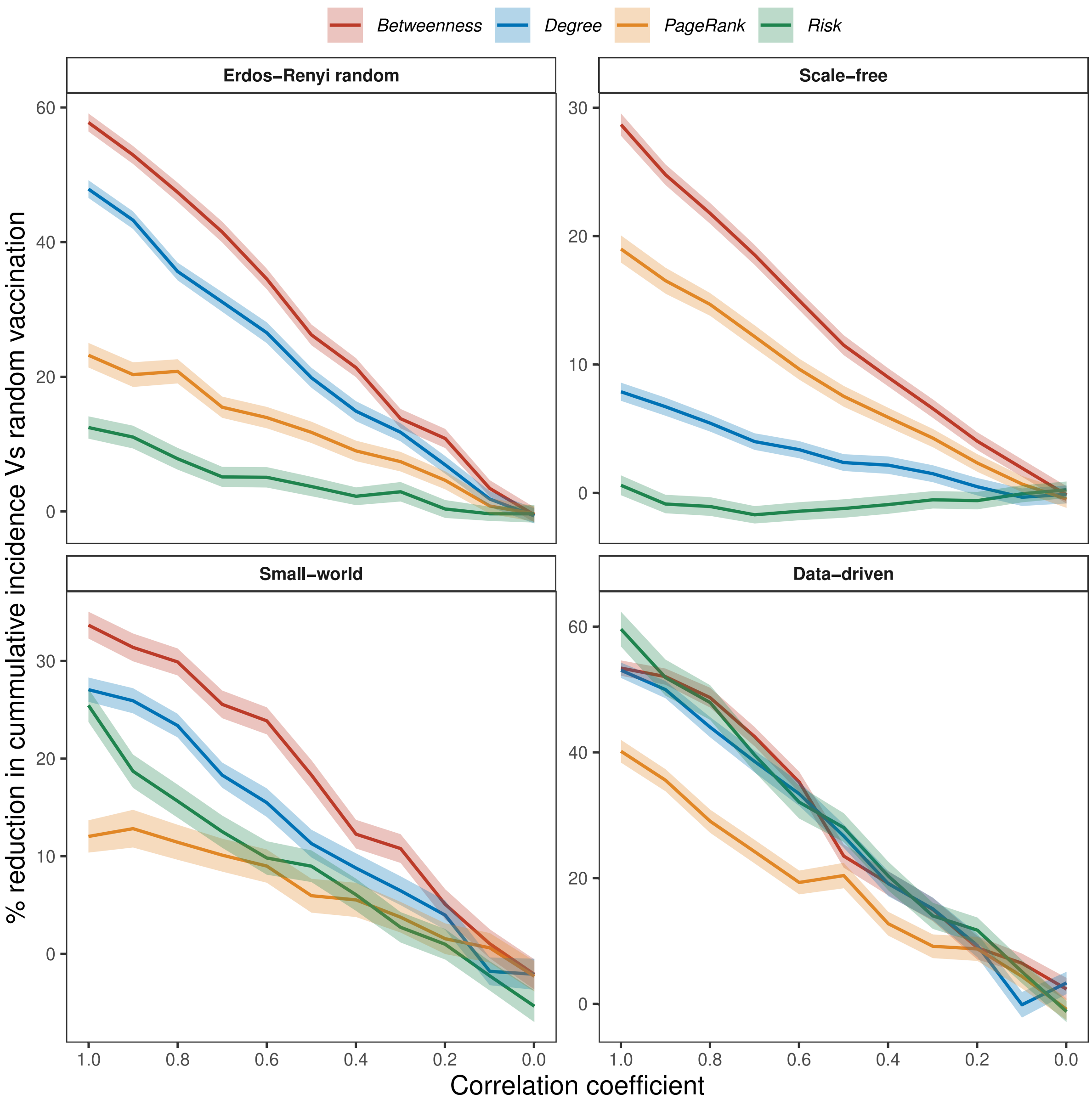

Figure S3. The effect of increasing noise on the effectiveness of vaccination strategies when 30% of nodes are vaccinated, at 75% within-node coverage

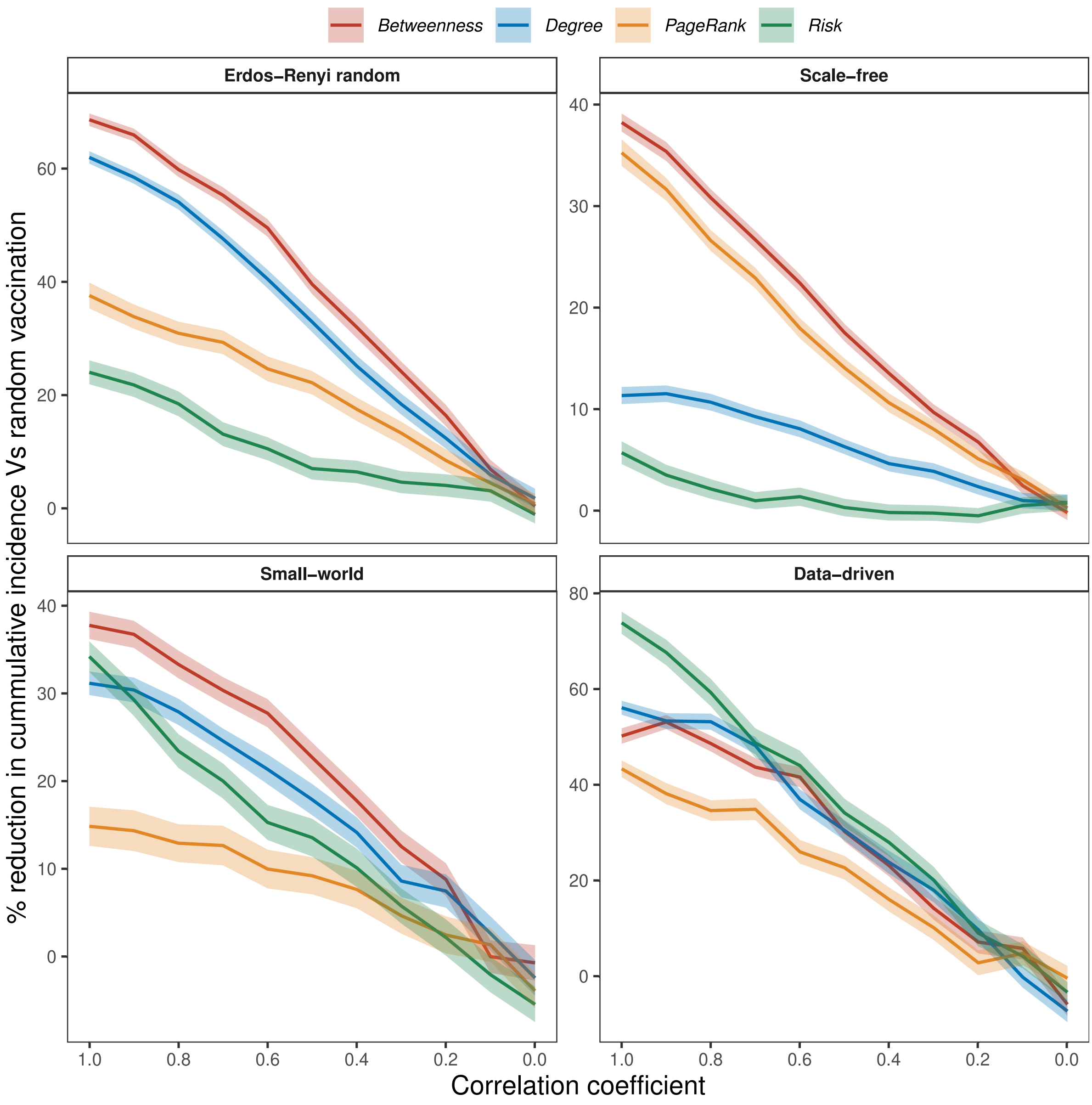

Figure S4. The effect of increasing noise on the effectiveness of vaccination strategies when 40% of nodes are vaccinated, at 75% within-node coverage

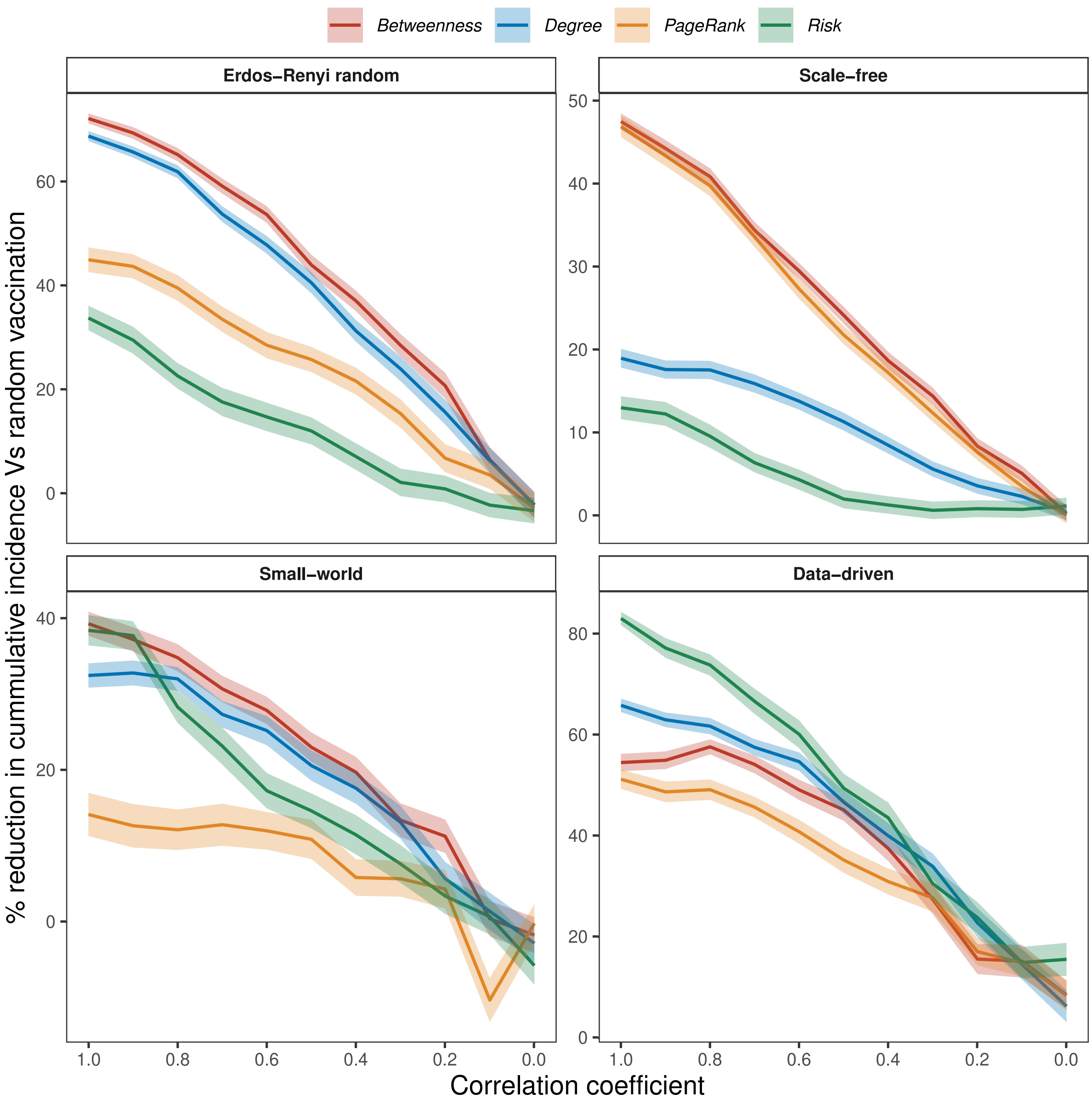

Figure S5. The effect of increasing noise on the effectiveness of vaccination strategies when 50% of nodes are vaccinated, at 75% within-node coverage

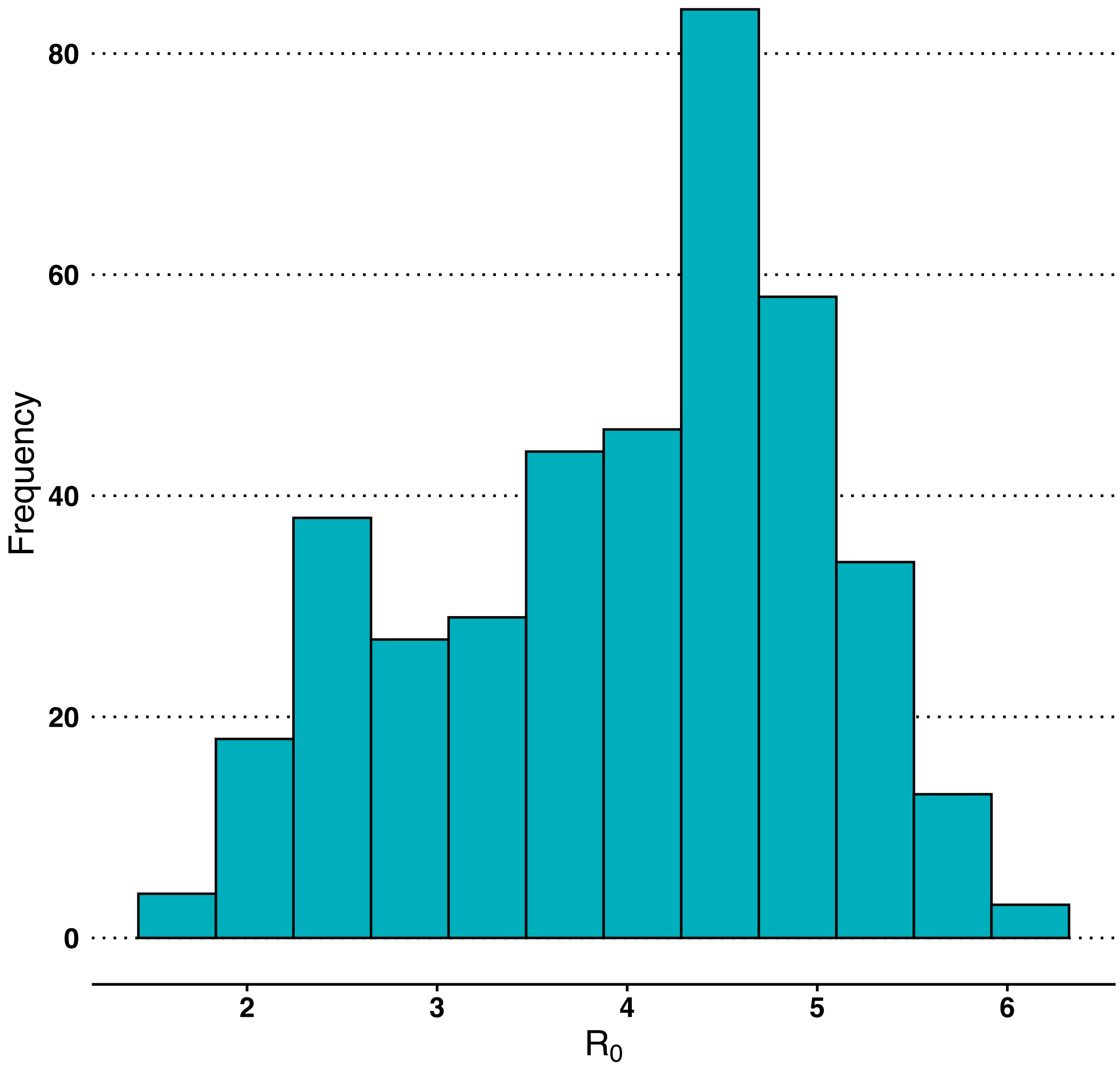

Figure S6. The distribution of R_0 values across 398 wards.

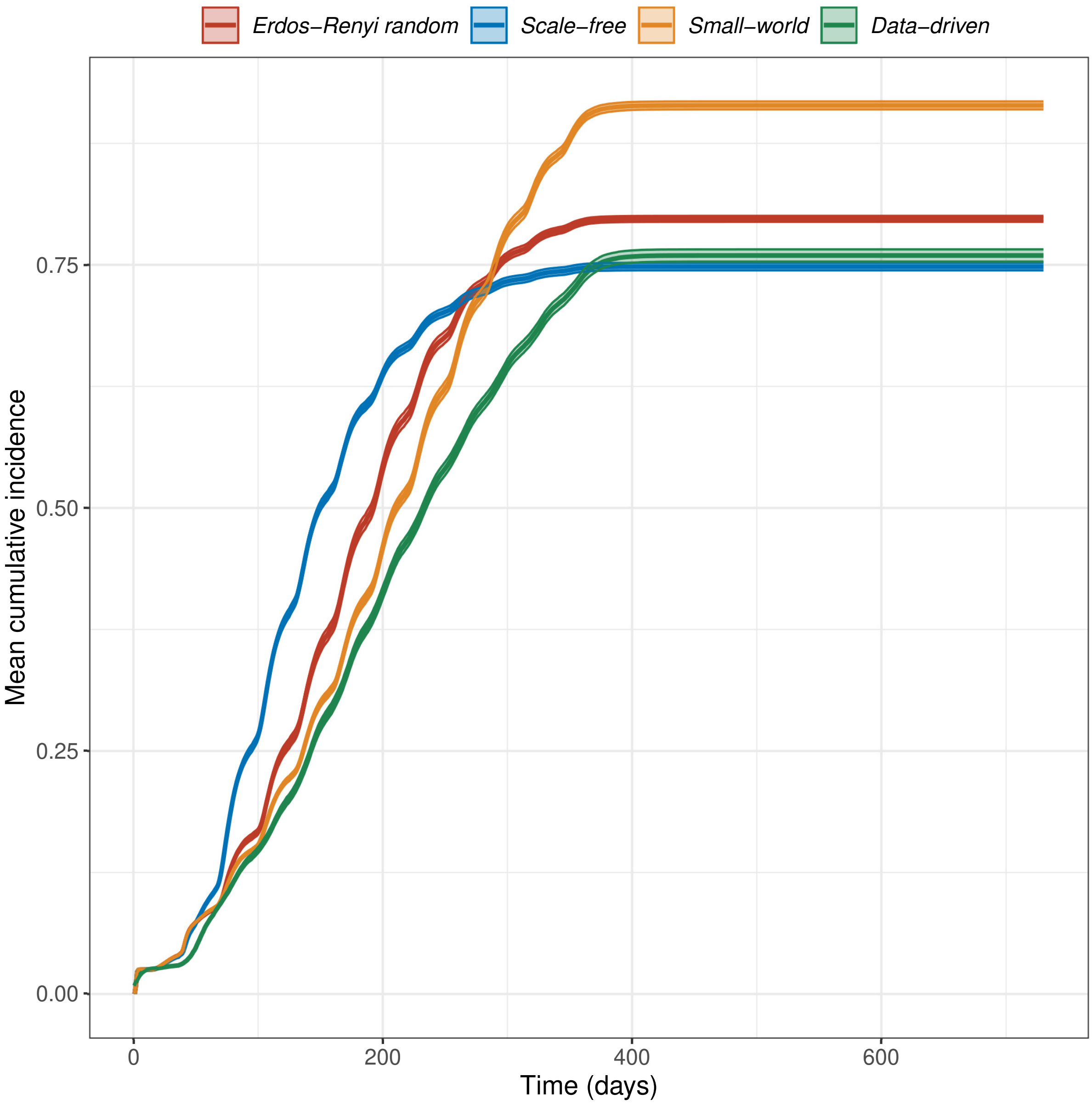

Figure S7. Mean cumulative incidence over 2 years for different network types. The plots shown represent the mean cumulative incidence of 400 simulations with a 95% confidence interval. All network types reached equilibrium before 500 days.

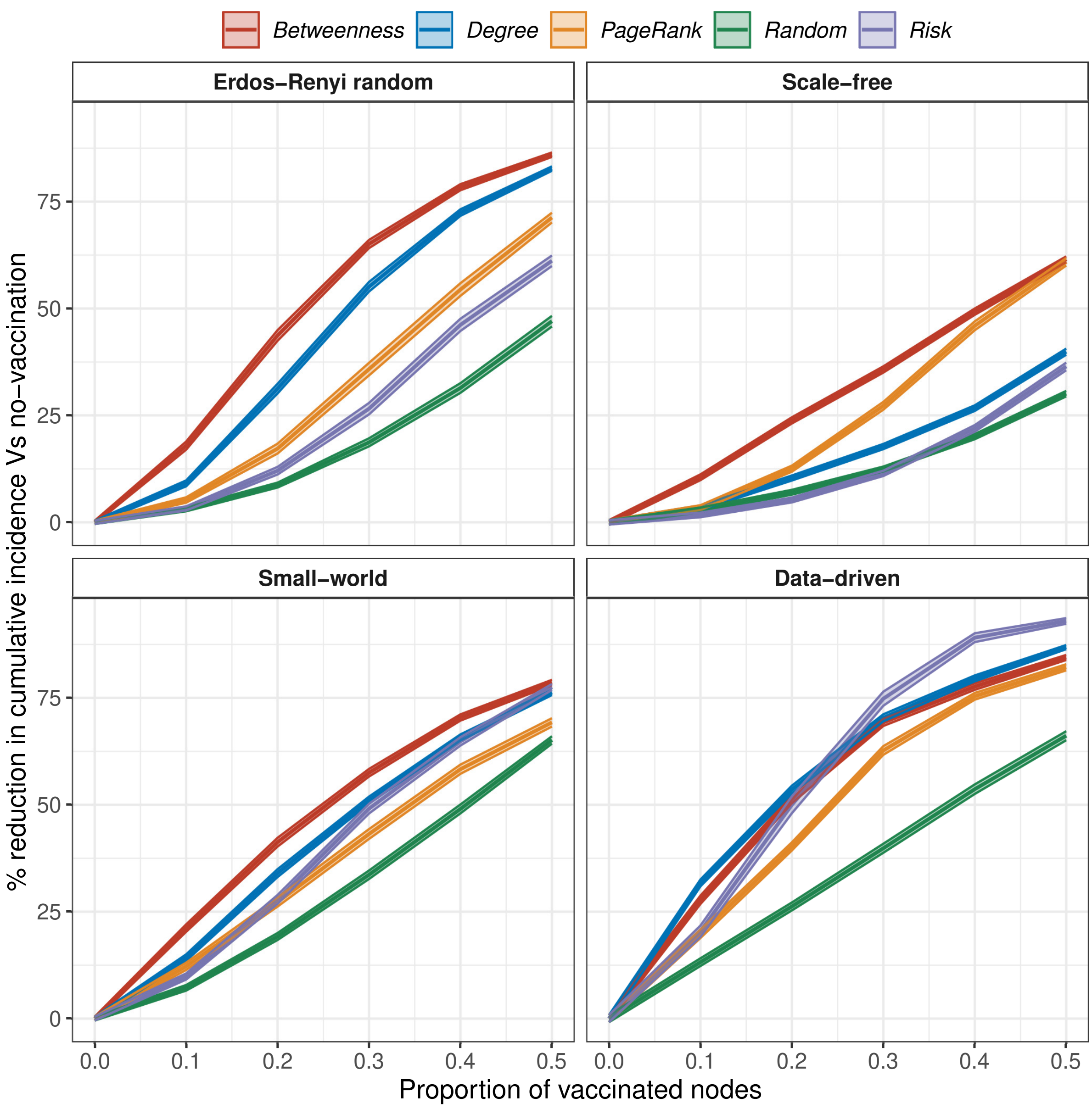

Figure S8. Effectiveness of vaccination strategies: Percentage reduction in node cumulative incidence with varying proportions of vaccinated nodes, at 75% within-node coverage. Simulations were conducted until the cumulative incidence reached an equilibrium state to compare the long-term effectiveness of targeted strategies with short-term results. The results from the extended simulation align with the findings presented in the main text.
